# Use of machine learning to identify patients at risk of sub-optimal adherence: study based on real-world data from 10,929 children using a connected auto-injector device

**DOI:** 10.1186/s12911-022-01918-2

**Published:** 2022-07-06

**Authors:** Amalia Spataru, Paula van Dommelen, Lilian Arnaud, Quentin Le Masne, Silvia Quarteroni, Ekaterina Koledova

**Affiliations:** 1 Swiss Data Science Center, ETH Zürich and EPFL, Zürich, Switzerland; 2 The Netherlands Organization for Applied Scientific Research TNO, P.O. Box 2215, 2301 CE Leiden, The Netherlands; 3 Connected Health and Devices, Global Healthcare Operations, Ares Trading S.A., An Affiliate of Merck KGaA, Eysins, Switzerland; 4 Global Medical Affairs Cardiometabolic & Endocrinology, Merck Healthcare KGaA, Darmstadt, Germany

**Keywords:** Adherence, Auto-injector, Connected device, Digital health, Indicator, Machine learning, Recombinant human growth hormone

## Abstract

**Background:**

Our aim was to develop a machine learning model, using real-world data captured from a connected auto-injector device and from early indicators from the first 3 months of treatment, to predict sub-optimal adherence to recombinant human growth hormone (r-hGH) in patients with growth disorders.

**Methods:**

Adherence to r-hGH treatment was assessed in children (aged < 18 years) who started using a connected auto-injector device (easypod™), and transmitted injection data for ≥ 12 months. Adherence in the following 3, 6, or 9 months after treatment start was categorized as optimal (≥ 85%) versus sub-optimal (< 85%). Logistic regression and tree-based models were applied.

**Results:**

Data from 10,929 children showed that a random forest model with mean and standard deviation of adherence over the first 3 months, infrequent transmission of data, not changing certain comfort settings, and starting treatment at an older age was important in predicting the risk of sub-optimal adherence in the following 3, 6, or 9 months. Sensitivities ranged between 0.72 and 0.77, and specificities between 0.80 and 0.81.

**Conclusions:**

To the authors’ knowledge, this is the first attempt to integrate a machine learning model into a digital health ecosystem to help healthcare providers to identify patients at risk of sub-optimal adherence to r-hGH in the following 3, 6, or 9 months. This information, together with patient-specific indicators of sub-optimal adherence, can be used to provide support to at-risk patients and their caregivers to achieve optimal adherence and, subsequently, improve clinical outcomes.

**Supplementary Information:**

The online version contains supplementary material available at 10.1186/s12911-022-01918-2.

## Background

The development of recombinant human growth hormone (r-hGH) has provided a more readily available treatment for growth disorders, although at a higher cost than human pituitary-derived growth hormone (GH), which was withdrawn over safety issues [1]. r-hGH is currently approved in many countries for the treatment of short stature associated with GH deficiency (GHD), Turner syndrome (TS), Prader–Willi syndrome (PWS), chronic renal insufficiency (CRI), short stature homeobox-containing gene deficiency (SHOX-D), and being born small for gestational age (SGA) [2]. Treatment with r-hGH involves daily subcutaneous injections, often for many years, to enable the children affected by these conditions to reach an adult height within or at least close to the normal range. Treatment success has been defined as doubling of pretreatment growth velocity after 12 months of treatment; close correlations have been observed between the calculated height velocity after 3 months and the observed height velocity after 12 months [3]. In addition to increasing final adult height, treatment with r-hGH also improves body composition and metabolism, decreasing visceral adipose tissue and improving lipid profiles [2, 4].

An important factor that determines the response to r-hGH treatment is adherence to the injection regimen [5, 6]. Optimal adherence is defined as a minimum of 85% of doses administered, equivalent to missing no more than one injection per week [7]. Motivation to adhere to treatment may reduce over time, partly because the benefits of r-hGH treatment are not immediately apparent, and also because daily subcutaneous injections present a significant burden to the children and their parents/caregivers [4]. The most obvious effects of poor adherence to r-hGH treatment are reductions in growth rates and final adult height [5, 7], but it can also result in wasted medication and increased healthcare costs [5, 8].

Whilst poor adherence is a well-recognized problem, accurate measurement of adherence to r-hGH treatment has always been difficult [4, 9]. Historically, unreliable proxy methods have had to be used, such as patient recollection, tracking prescriptions filled, or vials counted. Patients and parents/caregivers may be reluctant to admit to missing doses or do not remember accurately and may, therefore, overestimate their adherence to treatment when questioned by their healthcare providers (HCPs). Medication from prescriptions that are filled may not be used fully or may not be used at all [8]. Furthermore, supposedly objective methods such as vial counting do not indicate whether or not the medication has actually been used. Estimates of the prevalence of non-adherence range from 5 to 82% [10], demonstrating the great variability in the accuracy of methods used to evaluate and define adherence.

**Fig. 1:**
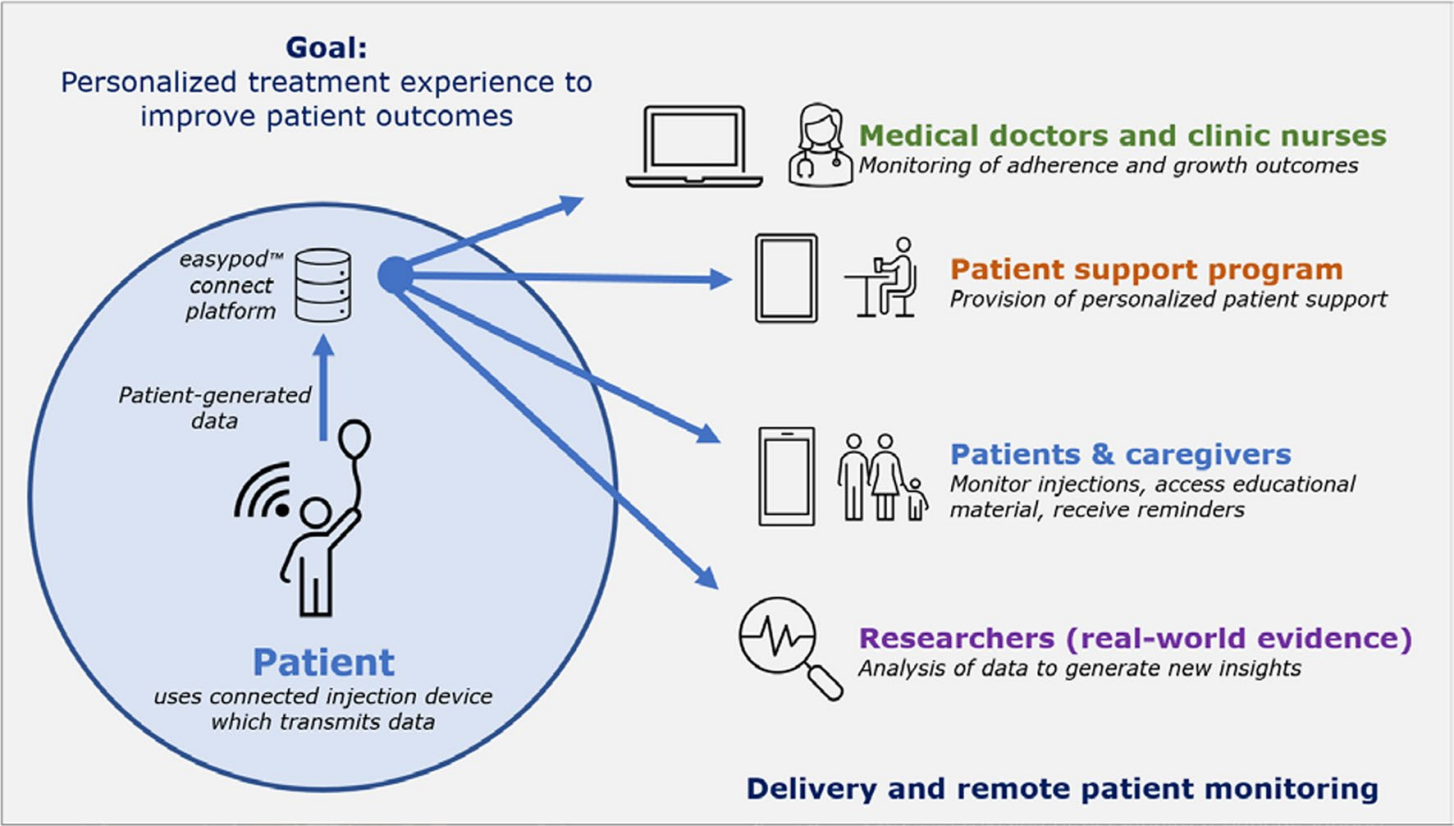
The easypod™ connect ecosystem for remote monitoring of therapy

This situation has now been improved, however, by the development of an electromechanical auto-injector device for r-hGH (easypod™; Merck Healthcare KGaA, Darmstadt, Germany) and a connected ecosystem (easypod™ connect), comprising a transmitter, software, and a secure, cloud-based data storage system (Fig. 1). The use of this ecosystem enables adherence to be assessed objectively using real-world data obtained from this connected device [11]. Similar digitally enhanced devices and systems have been utilized effectively in diabetes and asthma management in recent years [12–14]. The device accurately records the date, time, and actual dose administered along with the various comfort settings for the injector, such as injection speed and duration. The patient can then use the transmitter to send these data to their HCP and to the secure database. With access to the data, the HCP can review each individual patient’s level of adherence over time, giving them the opportunity of intervening, if necessary, with personalized corrective measures to try to improve adherence and subsequent outcomes. In addition, researchers can study the anonymized data stored in the database to analyze adherence behavior at the population level. This has demonstrated how important adherence is for optimal results following r-hGH treatment [15].

The aim of this study was to attempt the first integration (to the authors’ knowledge) of a machine learning approach into a digital health ecosystem to develop a model based on data from the first 3 months of treatment with r-hGH to identify early indicators and predict sub-optimal adherence (< 85%) over the following 3, 6, and 9 months using information obtained via the connected easypod™ device. The 85% threshold corresponds to approximately one missed injection per week [7], and below this value, the effectiveness of the treatment is known to be significantly affected [5, 8].

## Methods

### Patients

The study evaluated real-world data generated by patients registered or and transferring injection data using the easypod™ connect digital health ecosystem worldwide. Inclusion criteria were: starting to use the system during childhood (< 18 years old) and having a period of at least 12 months (1 year) between the first and the last recorded injection between 2007 and April 2020. Our research focused on the implementation component within the Ascertaining Barriers to Compliance (ABC) taxonomy of medication adherence [16].

Ethics approval was not required for this analysis, since we used secondary anonymized data from a commercially available service that collected data outside the scope of this study. The aforementioned commercial service had received all necessary legal approvals to allow secondary research purposes. The data was collected and analyzed according to the informed consent which was obtained at the time of data collection by the commercial service and provided without a patient identifier. The informed consent specifically allowed the use of data for secondary analysis and it was locally approved by legal teams and Data Privacy Officers.

### Data processing and feature engineering

For analysis purposes, data were extracted for all patients who recorded their injections and their injection settings in the auto-injector device during the period from 2007 to April 2020. The device automatically calculated and recorded the weekly adherence for a given week, based on the injected versus prescribed dose during that week. Data were then transmitted to the easypod™ connect database at the convenience of the patient/caregiver via wireless transmission from the easypod™ docking station to the cloud-based data storage system. The transmission dates, together with records of the injections given and other specific information (weekly adherence, injection settings, personal information), were used to calculate and create relevant features for each patient.

Features considered for the first 3 months of treatment were: number of transmissions, number of prescribed dose changes, most frequently used comfort settings (injection speed, injection depth, needle speed–which can be adjusted by a HCP according to patient preference–and injection time i.e. the duration for which the needle remains in the skin), mean weekly adherence (mean value of the weekly adherence records), weekly adherence standard deviation (SD; modeling week-to-week regularity), and personal information such as the patient’s gender and age at start of use.

### Study variable

The target variable to be predicted was the level of mean adherence during the following 3, 6, and 9 months after treatment start with respect to the 85% threshold. The task was, therefore, framed as a classification problem, where the positive class is “sub-optimal” adherence (< 85%) and the negative class “optimal” adherence (≥ 85%).

### Train-test split

The initial dataset of patients was unbalanced from the perspective of the two classes. In all three timeframes considered for prediction (3, 6, and 9 months), 78 to 80% of the patients had an optimal adherence level, while only 20 to 22% of patients had a sub-optimal level. There are several common techniques for working with machine learning applied to unbalanced datasets; these include resampling the dataset by over-sampling the minority class or under-sampling the majority class to achieve balance, reporting and optimizing for relevant performance metrics (e.g. F1-score, precision, sensitivity, specificity), or choosing appropriate algorithms [17]. To prevent biasing the algorithms towards the majority class (optimal adherence), we chose to train them on a balanced training dataset; however, to assess the algorithms’ performances under real-world conditions, the test set followed the real-life class distribution.

During our study, the negative class in the original dataset was first randomly under-sampled to achieve class balance, and the non-selected patients in the negative class were kept aside. Various models were trained on 80% of the balanced dataset and their hyper-parameters were optimized for the F1-score using a fivefold cross-validation scheme (for each of the three timeframes). The test set consisted of the remaining 20% of the balanced dataset, on to which we added the necessary number of previously non-selected negative class patients in order to achieve a class distribution similar to the original one: 78 to 80% negative class (optimal adherence) and 20 to 22% positive class (sub-optimal adherence). The optimized performance metric was the F1-score, which is defined as the harmonic mean between precision (the ratio between true positive predictions and all of the positive predictions) and recall (or sensitivity; the ratio between true positive predictions and the actual number of positive instances). The F1-score ranges between 0 and 1.

$$F1 - score = 2 \times \frac{Precision \times Recall}{{Precision + Recall}}$$

### Machine learning models and interpretation techniques

To predict adherence in a computationally efficient way, while also being able to interpret the prediction model in order to extract early indicators of near future adherence, we considered logistic regression and tree-based models [18–21]. The latter are widely used in biomedical applications due to their increased interpretability [12, 22]. Optimal/sub-optimal adherence in the following 3, 6, and 9 months was defined as target; and mean and SD adherence in the first 3 months, gender, age at start, number of transmissions, number of dose changes, the most frequently used injection time/speed/depth, and needle speed settings as predictors.

On the best performing model, global interpretation techniques based on SHapley Additive exPlanations (SHAP) values were applied [23–25] to identify the most important features for distinguishing between optimal and sub-optimal adherence, including the relation between a feature’s value and the model’s output (i.e. whether a high value drives predictions towards the positive or the negative class).

To obtain the typical thresholds used by the model for distinguishing between the two classes, local interpretation techniques [26] were applied on 10 randomly chosen instances of the optimal and sub-optimal classes, respectively. The aggregated thresholds were used to create Boolean features from continuous or multi-value features and their significance for distinguishing between the two classes was assessed by performing chi-squared tests [27]. The multiple testing problem was accounted for by further applying the Bonferroni correction [28] on the p-values associated with the chi-square test results (Additional file 1).

## Results

In total, 10,929 children aged < 18 years who started using the connected easypod™ device and transmitted injection data for ≥ 12 months were available for analysis**. Table ****1**** shows** the characteristics of the study population.

**Table 1:** Characteristics of the study population (N = 10,929)

Characteristics	Mean (SD) or P50 (P25– P75)	n (%)
Age at start (years)	9.7 (3.4)	
Gender		
Boys		6353 (58%)
Girls		4576 (42%)
Number of transmissions in first 3 months 0 ≥ 1		6042 (55%) 4887 (45%)
Adherence in first 3 months (%)	99 (94–100)	
Needle speed in first 3 months		
Slow		384 (4%)
Medium		5268 (48%)
Fast		5277 (48%)
Injection time in first 3 months (seconds)	8 (5–10)	
Injection depth in first 3 months		
4 mm		1424 (13%)
6 mm		6895 (63%)
8 mm		2248 (21%)
10 mm		362 (3%)
Number of dose changes in first 3 months 0 ≥ 1		8447 (23%) 2482 (77%)
Adherence < 85% 3 months		1964 (18%)
Adherence < 85% 6 months		2194 (20%)
Adherence < 85% 9 months		2419 (22%)

*SD* standard deviation

### Prediction performance

The four different machine learning models optimized on the train set in a fivefold cross-validation scheme included logistic regression [21], ordinal logistic regression [20], random forest [18], and extra trees [19]. Among these, the random forest model gave the best results in terms of average F1-score on the 5 cross-validation folds for all three prediction timeframes (3, 6, and 9 months) (Table 2).

**Table 2:** F1-scores for the four machine learning models evaluated over three time periods

Optimized Machine Learning Model	Mean (SD) F1-score (train set, fivefold cross-validation)		
	3 months prediction	6 months prediction	9 months prediction
Logistic regression	0.797 (0.012)	0.785 (0.016)	0.768 (0.012)
Ordinal logistic regression	0.798 (0.011)	0.784 (0.013)	0.768 (0.010)
Random forest	0.807 (0.012)	0.798 (0.018)	0.780 (0.011)
Extra trees	0.801 (0.017)	0.783 (0.021)	0.771 (0.01)

SD, standard deviation

**Fig. 2:**
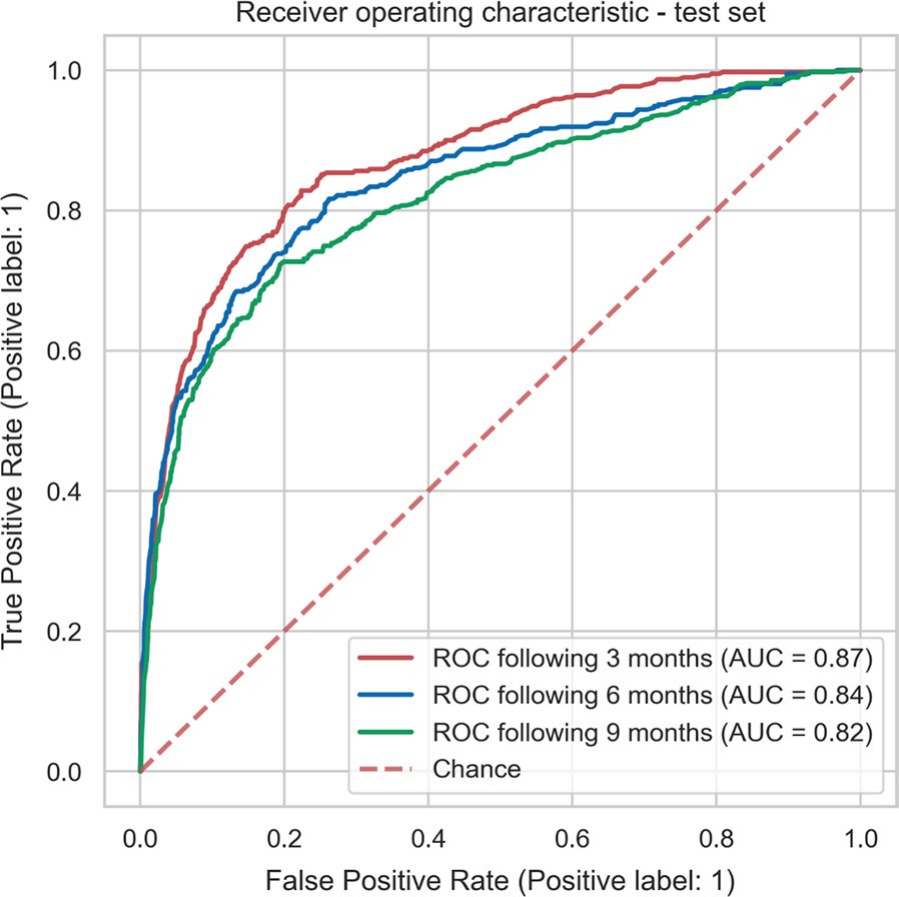
ROC curves to predict adherence to r-hGH therapy in the following 3, 6, and 9 months after starting r-hGH treatment. AUC, area under the curve; r-hGH, recombinant human growth hormone; ROC, receiver operating characteristic.

**Fig. 3:**
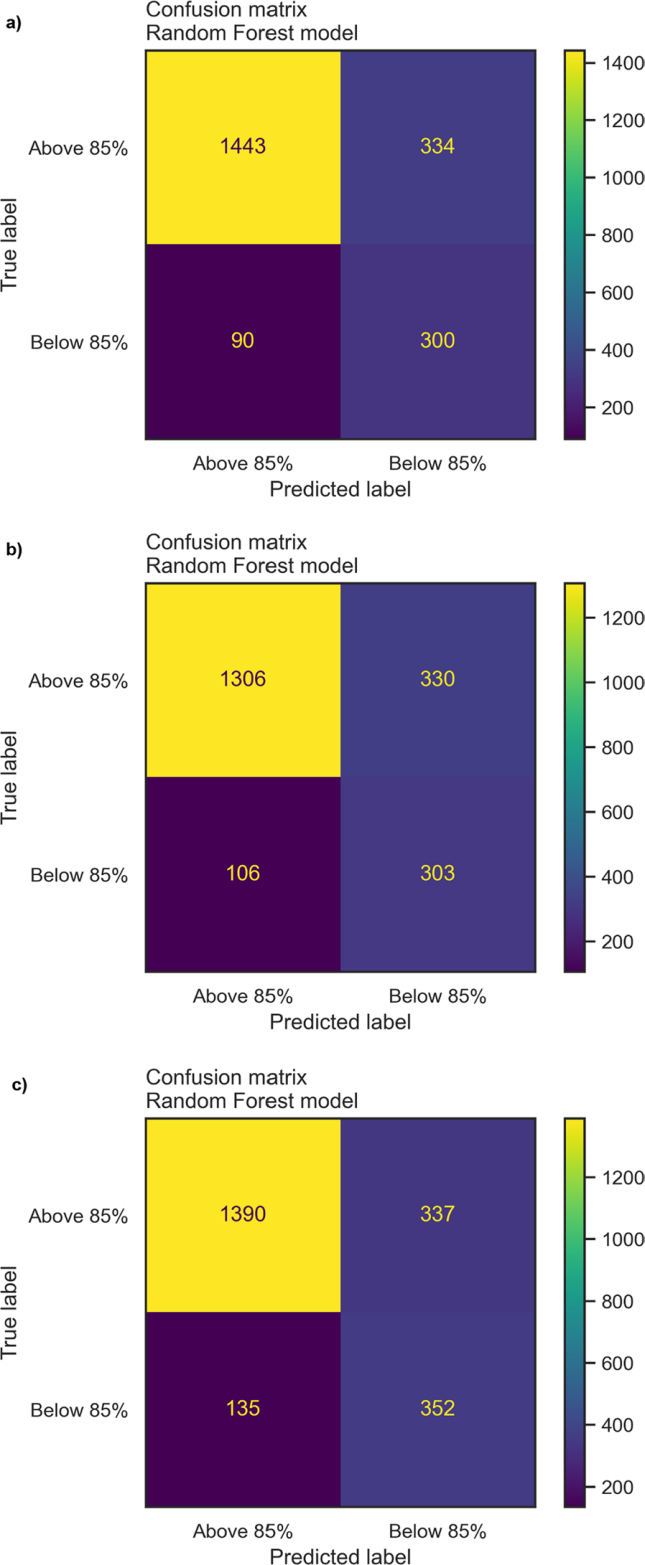
Random forest models to predict r-hGH therapy adherence in the following **a** 3, **b** 6, and **c** 9 months. The 85% threshold corresponds to approximately one missed injection per week; the effectiveness of treatment is known to be significantly affected below this value. r-hGH, recombinant human growth hormone.

The optimized random forest model was retrained on the entire balanced train set before being assessed on the unbalanced test set following a positive/negative class distribution similar to the real-world data (please refer to the *Train-test split* section). The model achieved an Area Under the Curve (AUC) between 0.82 and 0.87 for predicting sub-optimal mean adherence in the following 3, 6, and 9 months based on data in the first 3 months. **Figure ****2** shows the Receiver Operating Characteristic (ROC) curves with the random forest model for the three timeframes. **Figure ****3** shows the confusion matrices for the machine learning models to predict adherence for the three timeframes. The models achieved sensitivities between 0.72 and 0.77, specificities between 0.80 and 0.81, and F1-scores between 0.59 and 0.60 (Table 3).

**Table 3:** Random forest model versus simple heuristic baseline model performances over three time periods, reported on the test set

	3 months prediction		6 months prediction		9 months prediction	
	Random forest	Baseline	Random forest	Baseline	Random forest	Baseline
Sensitivity	0.77	0.49	0.77	0.44	0.72	0.39
Specificity	0.81	0.96	0.81	0.97	0.80	0.97
F1-score	0.59	0.60	0.59	0.57	0.60	0.52

### Indicators and predictors of adherence

**Fig. 4:**
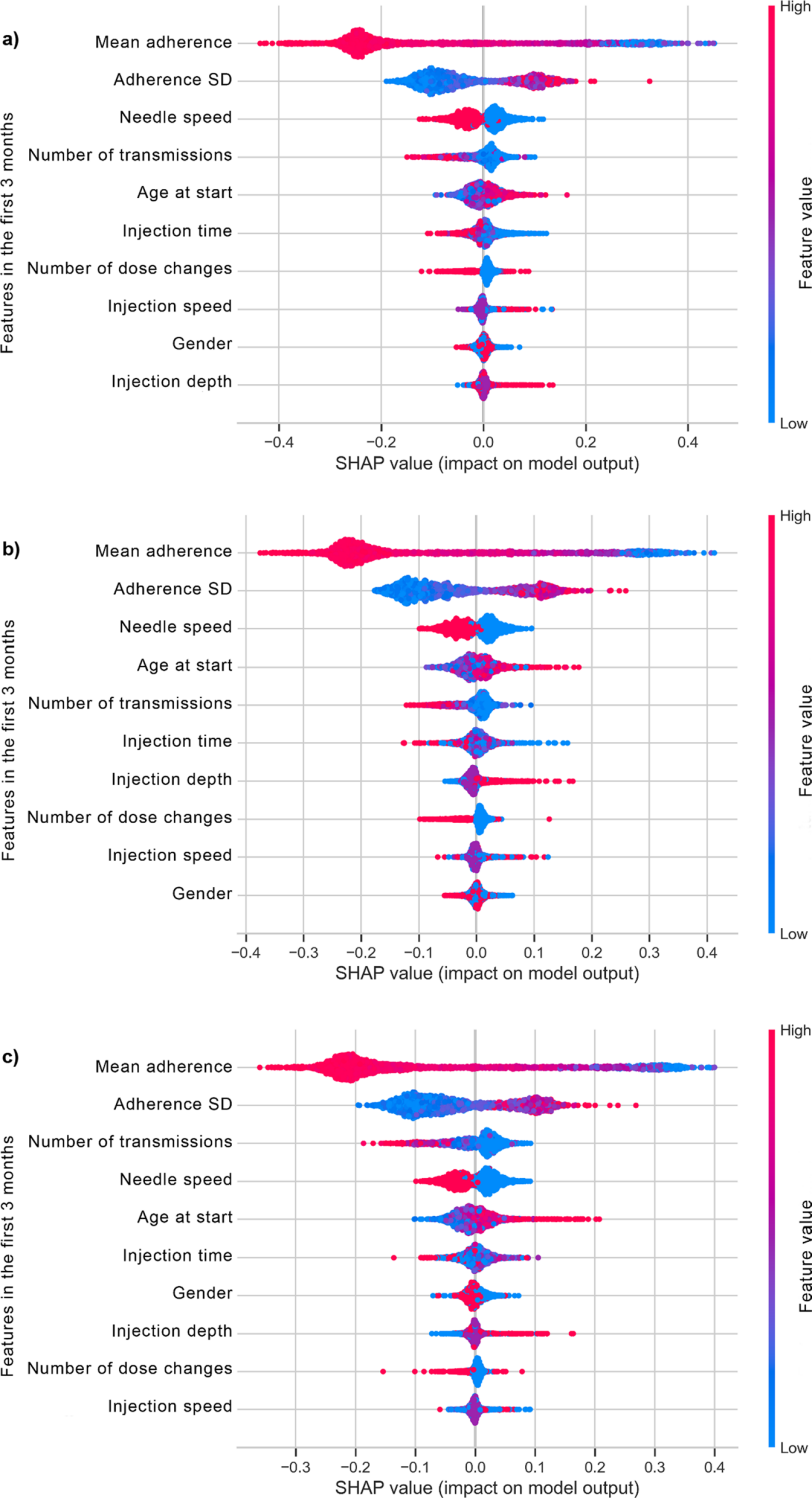
Relative importance of features to model output concerning r-hGH therapy adherence in the following **a** 3, **b** 6, and **c** 9 months after starting r-hGH treatment. SD, standard deviation; r-hGH, recombinant human growth hormone; SHAP, SHapley Additive exPlanations.

In the importance plot based on SHAP values with the random forest model (**Fig. ****4**), the features over the first 3 months are shown in decreasing order of importance for predicting the sub-optimal adherence class in the following months. The color map shows whether a high value on one particular feature drives model predictions towards the positive class (sub-optimal adherence, positive SHAP value) or the negative class (optimal adherence, negative SHAP value).

The adherence mean and SD over the first 3 months were the two most important features for predicting adherence in the following 3, 6, and 9 months: a high value of the mean adherence over the first 3 months drove model predictions towards the “optimal” class, while a high SD (high variability, low regularity) drove model predictions towards the “sub-optimal” class. When looking at the comfort settings for easypod™, the needle speed was the most important, and patients setting it to a high value (i.e. fast setting) had a lower risk of sub-optimal adherence than those setting it to a low value (i.e. slow setting).

Further interpreting the plot, we concluded that: (i) patients transmitting injection data more frequently were more likely to belong in the optimal adherence class; (ii) patients starting to use the connected auto-injector device at a later age had an increased risk of sub-optimal adherence; and (iii) a fast needle speed setting was a predictor of optimal adherence. Although very few patients had dose adjustments in the first 3 months, they tended to be on the optimal adherence side, and although gender was among the three features with the lowest importance, male gender (encoded as 1) drove the model’s prediction towards the sub-optimal class more than female gender (encoded as 0).

The Boolean features obtained as per the methodology described in the *Machine learning models and interpretation techniques* section [26] and which were significant under a Bonferroni-corrected [28] p-value assumption, are displayed in Table 4, together with their respective aggregated critical threshold. Injection speed and injection time were not statistically significant and, therefore, are not presented in Table 4.

**Table 4:** Boolean features which were significant under a Bonferroni-corrected p-value, together with their respective aggregated critical threshold

Feature in the first 3 months (with respect to a threshold value)	Bonferroni- corrected p-value		
	3 months prediction	6 months prediction	9 months prediction
Mean adherence < 90%	< 0.001	< 0.001	< 0.001
Adherence SD > 9%	< 0.001	< 0.001	< 0.001
Number of transmissions = 0	< 0.001	< 0.001	< 0.001
Fast needle speed	< 0.001	< 0.001	< 0.001
Age at start > 10 years old	< 0.001	< 0.001	< 0.001
No dose changes	0.002	< 0.001	–
Injection depth > 6 mm	0.021	< 0.001	0.002
Gender (male)	0.042	0.013	–

*SD* standard deviation

As we found that adherence over the first 3 months is the most significant predictor for adherence in the future, we compared our model to a simple heuristic model that would predict sub-optimal (respectively, optimal) adherence in the future if adherence is sub-optimal (respectively, optimal) over the first 3 months. The sensitivity, specificity, and F1-score (on the test set) of this simple model for predicting sub-optimal adherence in the following 3, 6, or 9 months were between 0.39 and 0.49, 0.96 and 0.97, and 0.52 and 0.60, respectively (Table 3).

## Discussion

We developed an accurate model predicting how likely a child’s adherence will be < or ≥ the optimal threshold (85%) over the following 3, 6, and 9 months, based on data from the first 3 months and early indicators of sub-optimal adherence to r-hGH therapy when using the connected easypod™ device. Due to the importance of both optimal adherence and the treatment starting phase for the success of treatment with r-hGH, the ability to not only predict which patients are at risk of sub-optimal adherence but also to recognize the early indicators is a valuable asset for the HCP teams managing the treatment of these young patients. Our research aimed to be the first attempt to design a machine learning approach integrated into a digital health ecosystem to manage adherence to and provide clinical decision support for children receiving GH therapy via a connected auto-injector device. Recent calls towards human-centered artificial intelligence (AI) with explainability features supporting HCPs to understand recommendations and make informed decisions have been raised in the literature [29]. For this present work, we focused on a data-driven design culture and the need to engage HCP teams to interact with the values provided by the digital health ecosystem. Indeed, our results can serve as an example for other ecosystems to show how AI techniques on real-world data can be used in daily practice to provide accurate and personalized advice based on a patient's historic treatment journey.

We employed a machine learning and statistics approach to conduct our study. The random forest model (the best performing model among the four different optimized models), besides being able to model non-linear data, has the advantage of being highly compatible with advanced machine learning interpretation techniques based on game theory SHAP values [23–25] or local linear estimations [26]. This, in turn, enabled the identification of the main adherence drivers–information that can be readily used by HCPs. SHAP-type visualizations can support the understanding of factors contributing to adherence which can ultimately support the creation of next-generation Clinical Decision Support Systems. In the absence of model predictions, adherence can be improved through measures including modification of comfort settings for the injection device such as changing the needle speed and the injection depth, encouraging transmissions, or evaluating whether a dose change is needed. While our study is a multi-variate analysis, which is by itself one way to control potential confounding factors, the injection depth and age may benefit a deeper investigation (e.g. through stratification), as adults typically require a deeper injection.

The demonstrated importance of the initial phase of 3 months of treatment with r-hGH [3], as well as a clinical need to identify early indicators and take action as soon as possible in case of undesired treatment behavior, underpinned our choice to build the models based on data from the first 3 months of treatment only. However, once the starting treatment phase is over, the model could be deployed on a continuous basis, by updating each month the future prediction based on the previous 3 months of data. Our study evaluated the prediction of optimal/sub-optimal adherence over three possible timeframes: the next 3, 6, or 9 months. While specificity and F1-scores remain similar, sensitivity was substantially higher when predicting short-term adherence behavior (0.77 or 77%, timeframe: 3 and 6 months) versus long-term adherence behavior (0.72 or 72%, timeframe: 9 months). Indeed, the longer the timeframe, the greater the probability of unexpected events that may alter injection adherence and, hence, the more unpredictable the sub-optimal adherence behavior. Furthermore, eight features were shown to be important for predicting future sub-optimal adherence, some of which were related to not using the system’s features (e.g. infrequent transmission of data and not changing some of the comfort settings, such as the needle speed setting) or starting treatment at an older age. We calculated the critical thresholds of all eight of these features in order to help HCPs to classify patients who may be at risk of future sub-optimal adherence. For example, patients aged more than 10 years at the start of their treatment had a statistically significantly (p < 0.001) higher risk of future sub-optimal adherence than patients who started treatment earlier. These patients are likely to require additional support to adhere to r-hGH therapy, on the basis of our results. Similarly, not transmitting data during the first 3 months of treatment with r-hGH was identified as a statistically significant indicator of sub-optimal adherence. This observation (which would be invaluable to HCPs) was only possible because we included data from patients with 0 transmissions within the first 3 months. However, actually performing a prediction for these patients would not be possible in a real-world setting because there would be no data at that time. In this case, and given the fact that the absence of transmissions suggests an increased risk of non-adherence and warrants follow-up, we can envision a system that notifies the HCP if a registered patient has not transmitted data within the first 3 months of their r-hGH treatment.

The specificity versus sensitivity trade-off would itself benefit from discussion between medical experts. The machine learning model outputs a class probability from 0 to 1 and the current sensitivities, specificities, and F1-scores mentioned in the *Prediction performance* section are calculated for a standard prediction probability threshold of 0.5, above which the model predicts sub-optimal adherence. The current specificity of 80–81% means that the model misclassifies optimal adherence as sub-optimal in ~ 20% of cases. In terms of absolute numbers, this translates into a number of false positive predictions close to that of true positive ones, meaning that HCPs would have to follow-up unnecessarily on almost half of the patients flagged as being at risk of sub-optimal adherence by the model. Specificity can be increased by choosing a different threshold on the ROC curve; however, this comes at the expense of a loss of sensitivity. The optimal point on the ROC curve may not be the same for everyone and it ultimately needs to answer an ever-recurring question: do HCPs need to identify as many patients at risk of sub-optimal adherence as possible and take a rather precautious approach, or should they rather avoid unnecessary workload and patient nudging?

Strengths of our study include user-centered design aiming to provide clinical decision support for children receiving r-hGH therapy via a connected auto-injector device and conducting rigorous testing using validation methods on a large study population from which accurate real-world data could be extracted by means of the connected easypod™ device. Examples of connected devices with data collection and data transmission capabilities have also been successfully utilized in other therapeutic areas, such as asthma and diabetes [13, 14], and examples of adoption of explainable AI–how to provide appropriate information to help users understand the AI’s functions and decisions –are described in the literature [29–31].

The same methodology can be replicated, therefore, to different medical conditions, provided that similar data are available. In fact, several machine learning approaches have already been used to successfully evaluate adherence in other therapeutic areas [32, 33].

This study aimed to derive indicators of and to predict sub-optimal adherence for persistent patients. However, non-persistence is a closely related problem which was not addressed here. By construction of the dataset, all considered patients had a persistence of at least 12 months. While this study did not evaluate whether non-persistent patients would be classified by the algorithms as having sub-optimal adherence, a previously performed study investigated predictors of non-persistence and modeled the risk of non-persistence over the following 6 months [34]. The two models could be used together for a more complete patient insight and to provide clinical support. Further work could evaluate whether the results of the two models are consistent, i.e. whether patients with a high risk of non-persistence are also classified as having sub-optimal adherence.

A limitation of our study included the fact that statistical tests on the difference in F1-scores between the models were not performed, which may have resulted in differences observed by chance. Furthermore, the F1-scores on the test set (0.59–0.60) were up to 0.20 lower with respect to the average ones obtained on the train set in a fivefold cross-validation scheme (0.78–0.80). The underlying reason is likely because the training set was a balanced one and, therefore, the model does not favour the optimal (negative) adherence prediction. The test set, however, followed a real-life unbalanced distribution with considerably more optimal adherence than sub-optimal adherence cases. Future work could compare results from this methodology with results from where the training set would follow the same distribution as the test set. Another secondary reason might be over-fitting on the training set, although cross-validation was applied; further refinements of the model could propose a train-validation-test schema instead.

In addition, dose change was only investigated during a short timeframe of 3 months; studies with a longer timeframe are needed to validate the impact of no dose change on sub-optimal adherence. Furthermore, previous research showed that both self-injection and age impacts sub-optimal adherence [35]. In this current study, we did not have data on whether it was the child who injected themselves or if it was their parent. Therefore, we were not able to investigate the interaction between age and self-injections on future sub-optimal adherence. Lack of data was also the reason why, when analyzing factors that are impacting adherence, several other potentially relevant variables such as cost, insurance status, or comorbidities were not assessed and included. This is one more limitation of the study and, upon availability, future work could consider enriching the dataset with this information, so that its impact is equally analyzed. Another limitation includes the fact that our study did not address the growth outcomes achieved by the sub-optimal and optimal adherence groups due to the lack of a bank of height measurement data equivalent to the adherence data. However, previous studies have shown that better adherence translates into better outcomes for the children involved [4, 6, 8, 36]. Further evaluation is required to assess performance of this model in terms of reliability, utility, and expandability as part of clinical decision support.

A final observation concerns the comparison of the machine learning model with the simple heuristic baseline model built on the assumption that future adherence is optimal/sub-optimal if adherence in the first 3 months is optimal/sub-optimal. The performance of this simple model increasingly declines in terms of both sensitivity and F1-score when the prediction timeframe increases from 3 to 6 months, and eventually to 9 months. The F1-score of the machine learning model does not differ substantially to that of the simple model for the short-term predictions, but reduces abruptly for the 9 months’ prediction while the specificity remains similar between the different prediction timeframes; specificity was higher for the simple model (0.96–0.97) than for the machine learning model (0.80–0.81). However, when comparing the two models based on sensitivity, the machine learning model (0.72–0.77) demonstrates a much higher performance over the simple one (0.39–0.49). We can therefore conclude that the simple model could be a good option for predicting optimal adherence and for reducing the risk of false sub-optimal adherence alarms, although this approach can only identify < 50% of the patients with actual future sub-optimal adherence. Referring to the sensitivity–specificity trade-off discussed earlier, future work could investigate how these performances change when setting a different adherence threshold for the first 3 months for the simple model. However, not only do the additional features in the machine learning model enhance the sensitivity rate and give insights on other factors impacting adherence (e.g. weekly adherence consistency, transmission and use of the e-device and its settings), re-training it regularly in a production environment would enable this model to account for shifts in the adherence behaviour of patients over time which could not be taken into account when a fixed threshold is considered. Even in the case where a simple model could satisfy the performance requirements, these last points are arguments in favour of using a more complex machine learning model and warrant a discussion between the medical and technical professionals when deciding on the adoption of such a predictive model.

## Conclusions

Our study emphasizes the power of AI techniques–big data processing, machine learning, and statistical models–on real-world data from more than 10,000 patients with growth disorders to predict future adherence to r-hGH, with sensitivities of between 72 and 77% and specificities of between 80 and 81%. Infrequent transmission of data, certain comfort setting values, and starting treatment at an older age were regarded as major factors in predicting the risk of sub-optimal adherence in the following 3, 6, or 9 months after starting r-hGH treatment.

Real-world data enables accurate predictions of clinically meaningful and explainable outcomes related to GH treatment. which can be deployed in clinical practice using digital health platforms. This allows HCPs to personalize therapy at any stage of their patients’ journey and improve shared decision-making with both patients and caregivers to achieve optimal growth outcomes.

In addition to enabling personalized healthcare and improving patient–HCP communication, our model has demonstrated the association between a good level of adherence to treatment with r-hGH and being engaged with the electronic drug-delivery system, i.e. transmitting frequently and changing the comfort settings. This latest finding generated a new research question: is engaging with a digital ecosystem per se improving treatment-related metrics such as adherence, persistence of use and growth outcomes? The results of this work are, however, outside the scope of the current paper.

Lastly, for machine learning models to fully open the path to personalized healthcare and patient support, further research is needed to investigate their feasibility, acceptability, and expandability to guide and improve clinical decisions.

## Supplementary Information

**Additional file 1**. Modeling code.

## Data Availability

Any requests for data by qualified scientific and medical researchers for legitimate research purposes will be subject to Merck’s Data Sharing Policy. All requests should be submitted in writing to Merck’s data sharing portal https://www.merckgroup.com/en/research/our-approach-to-research-and-development/healthcare/clinical-trials/commitment-responsible-data-sharing.html. When Merck has a co-research, co-development, or co-marketing or co-promotion agreement, or when the product has been out-licensed, the responsibility for disclosure might be dependent on the agreement between parties. Under these circumstances, Merck will endeavor to gain agreement to share data in response to requests.

## Abbreviations

AUC: Area under the curve
CRI: Chronic renal insufficiency
GH: Growth hormone
GHD: Growth hormone deficiency
HCP: Healthcare provider
PWS: Prader–Willi syndrome
r-hGH: Recombinant human growth hormone
ROC: Receiver operating characteristic
SD: Standard deviation
SGA: Small for gestational age
SHAP: SHapley Additive exPlanations
SHOX: Short stature homeobox-containing gene deficiency
TS: Turner syndrome
