# Supplementary material for: Use of machine learning to identify patients at risk of sub-optimal adherence: study based on real-world data from 10,929 children using a connected auto-injector device
Source: BMC Med Inform Decis Mak. 2022 Jul 6;22:179. doi: 10.1186/s12911-022-01918-2 (PMC9261072; doi:10.1186/s12911-022-01918-2)
Supplement: Supplementary file 1 — Additional file 1. Modeling code. [file 12911_2022_1918_MOESM1_ESM.docx]

**Supplementary File**

**Use of machine learning to identify patients at risk of sub-optimal adherence: Study based on real-world data from 10,929 children using a connected auto-injector device**

**Amalia Spataru^1^, Paula van Dommelen^2^*, Lilian Arnaud^3^, Quentin Le Masne^3^, Silvia Quarteroni^1^, Ekaterina Koledova^4^**

*^1^Swiss Data Science Center, ETH Zürich and EPFL, Zürich, Switzerland; ^2^The Netherlands Organization for Applied Scientific Research TNO, Leiden, the Netherlands; ^3^Connected Health & Devices, Global Healthcare Operations, Ares Trading SA, Eysins, Switzerland, an affiliate of Merck KGaA; ^4^Global Medical Affairs Cardiometabolic & Endocrinology, Merck Healthcare KGaA, Darmstadt, Germany*

**Modeling code**

conda install -c conda-forge mord

conda install -c conda-forge/label/cf202003 mord

conda install spyder-notebook -c spyder-ide

pip install shap

pip install lime

pip install mysql-connector-python-rf

import webbrowser

# define global variable

PATH_DATA = "/data/data_1year_adhpred.csv"

PATH_WEEKLYADH = "/data/WEEKLY_ADHERENCE_WITH_HEADER_FINAL.csv"

# import packages

%reload_ext autoreload

%autoreload 2

from datetime import date, datetime, timedelta

import json

import pickle as pkl

import os

import re

import numpy as np

import pandas as pd

from time import time

from tqdm import tqdm

from dateutil.relativedelta import *

import mysql.connector as connect

import matplotlib.pyplot as plt

import matplotlib.patches as mpatches

import matplotlib.lines as mlines

import matplotlib.cm as cm

import seaborn as sns

from sklearn.pipeline import Pipeline

from sklearn.preprocessing import PowerTransformer, MinMaxScaler, StandardScaler, RobustScaler

from sklearn.model_selection import train_test_split, GridSearchCV, StratifiedKFold

from sklearn.feature_selection import RFECV

from sklearn.linear_model import LogisticRegression

from sklearn.tree import DecisionTreeClassifier

from sklearn.ensemble import RandomForestClassifier, ExtraTreesClassifier

from sklearn import metrics

from sklearn.metrics import plot_confusion_matrix

from sklearn.metrics import confusion_matrix

import mord

#model intepretation

import shap

import lime

import lime.lime_tabular

# statistical tests

from scipy.stats import chi2_contingency

from statsmodels.sandbox.stats.multicomp import multipletests

#Add additional adherence periods

newdf = pd.read_csv(

PATH_WEEKLYADH,

sep = ",",

index_col=False,

# rename accordingly (problem with names vs number of columns)

names = [

"PATIENT_ID","YEAR_OF_WEEK","WEEK_OF_YEAR",

"START_DATE","END_DATE","DOSE_ADHERENCE",

"AVG_PRESC_DOSE",

"AVG_PRESC_DOSE_FREQ","STDDEV_DOSE_ADHERENCE",

"MEAN_INTERVAL_BETWEEN_INJECTIONS",

"STDDEV_DURATION_SINCE_LAST_INJECTION",

"TIME_IN_HOURS_SINCE_MIDNIGHT",

"STDDEV_TIME_IN_HOURS_SINCE_MIDNIGHT",

"WEEK_NR","DATE_OFF",

"DOSE_DAY_1","DOSE_DAY_2","DOSE_DAY_3","DOSE_DAY_4","DOSE_DAY_5","DOSE_DAY_6","DOSE_DAY_7",

"DAY_WITH_INJECTIONS","NB_INJECTIONS"],

skiprows=1

)

# preprocessing block

# keep only certain useful columns

touse = ["PATIENT_ID", "YEAR_OF_WEEK", "WEEK_OF_YEAR", "START_DATE", "END_DATE", "WEEK_NR",

"DOSE_ADHERENCE", "AVG_PRESC_DOSE_FREQ", "DAY_WITH_INJECTIONS", "NB_INJECTIONS"]

newdf = newdf[touse]

# keep only weeeks 1 to 52

newdf = newdf[newdf["WEEK_NR"].between(1,52)]

newdf.loc[((newdf["DOSE_ADHERENCE"].isnull()) & (newdf["NB_INJECTIONS"] == 0)), "DOSE_ADHERENCE"] = 0

remp = newdf[newdf["DOSE_ADHERENCE"].isnull()]["PATIENT_ID"].unique().tolist()

newdf = newdf[~newdf["PATIENT_ID"].isin(remp)]

newdf["DOSE_ADHERENCE"] = newdf["DOSE_ADHERENCE"]*100 #transform adherence to 100% scale

df_adhsecond = newdf[newdf["WEEK_NR"].between(14,26)] #weeks 14 - 26

df_adhthird= newdf[newdf["WEEK_NR"].between(14,39)] #weeks 14 - 39

df_adhfourth = newdf[newdf["WEEK_NR"].between(14,52)] #weeks 14 - 52

df_adhsecond = (

df_adhsecond.groupby("PATIENT_ID")["DOSE_ADHERENCE"]

.agg(DOSE_ADHERENCE3m="mean")

.reset_index())

df_adhthird = (

df_adhthird.groupby("PATIENT_ID")["DOSE_ADHERENCE"]

.agg(DOSE_ADHERENCE6m="mean")

.reset_index())

df_adhfourth = (

df_adhfourth.groupby("PATIENT_ID")["DOSE_ADHERENCE"]

.agg(DOSE_ADHERENCE9m="mean")

.reset_index())

newdf2 = df_adhsecond.merge(df_adhthird , left_on="PATIENT_ID", right_on="PATIENT_ID")

newdf3 = newdf2.merge(df_adhfourth , left_on="PATIENT_ID", right_on="PATIENT_ID")

# read pre-saved data

df = pd.read_csv(PATH_DATA).drop(["BIRTH_YEAR", "START_DATE"], axis=1)

df.sort_values(by=['PATIENT_ID'])

#merge with other periods of future adherence

print(len(df.index))

df = df.merge(newdf3 , left_on="PATIENT_ID", right_on="PATIENT_ID")

print(len(df.index))

def feature_distributions(df, cols_to_plot):

# print main statistics

display(df[cols_to_plot].describe(percentiles=[.01,.25, 0.5, .75, .95, .99, .997]).round(1))

print("\n")

# plot distributions

sns.set(context="notebook")

df[cols_to_plot].hist(bins=50, layout=(4,4), figsize = (21, 12))

plt.show()

def return_rescaled_data(df, cols_to_transform):

pt = PowerTransformer()

df_transf = df.copy()

df_transf[cols_to_transform] = pt.fit_transform(df_transf[cols_to_transform])

return df_transf

df.columns

# analyzing and plotting distributions of continous variables

toplot =["AGE_START", "DOSE_ADHERENCE", "ADH_MEAN",

"ADH_MAX", "ADH_MIN", "ADH_STD", "DOSE_ADHERENCE3m",

"DOSE_ADHERENCE6m", "DOSE_ADHERENCE9m","NR_TRANS", "NR_DOSE_CHANGES",

"INJECTION_TIME"]

feature_distributions(df, cols_to_plot=toplot)

# removing outliers

df = df[

(df["DOSE_ADHERENCE"].between(0,110))

&(df["ADH_MEAN"].between(0,110))

&(df["ADH_STD"].between(0,110))

&(df["DOSE_ADHERENCE3m"].between(0,110))

&(df["DOSE_ADHERENCE6m"].between(0,110))

&(df["DOSE_ADHERENCE9m"].between(0,110))

]

# cutting at 17 (based on distribution of transmission number and anomalously high nr of dose changes in some cases)

df = df[

(df["NR_TRANS"] < 17)

&(df["NR_DOSE_CHANGES"] < 17)

]

# analyzing and plotting distributions of continous variables

toplot =["AGE_START", "DOSE_ADHERENCE", "ADH_MEAN",

"ADH_MAX", "ADH_MIN", "ADH_STD", "DOSE_ADHERENCE3m", "DOSE_ADHERENCE6m",

"DOSE_ADHERENCE9m","NR_TRANS", "NR_DOSE_CHANGES",

"INJECTION_TIME"]

feature_distributions(df, cols_to_plot=toplot)

# print data shape after cleaning

print("Number of patients:", df.shape[0])

print("Number of features:", df.shape[1])

#Number of patients: 10929

#Number of features: 20

#recalling what are the columns in the dataframe

df.columns

# plot a heatmap with the absolute value of pearson's correlation between continuous features

cat_var = ["GENDER", "INJECTION_SPEED", "INJECTION_DEPTH", "NEEDLE_SPEED", "HSDS_START_LOW"]

corr_pearson = df.drop(cat_var, axis=1).corr(method="pearson")

# plot correlation matrix

sns.set(style="white")

# Generate a mask for the upper triangle

mask = np.triu(np.ones_like(corr_pearson, dtype=np.bool))

# Set up the matplotlib figure

f, ax = plt.subplots(figsize=(12, 9.5))

# Generate a custom diverging colormap

cmap = sns.diverging_palette(240, 10, n=20)

# Draw the heatmap with the mask and correct aspect ratio

ax = sns.heatmap(corr_pearson.abs(), mask=mask, cmap=cmap, vmin=0.0, vmax=1.0, center=0,

square=True, linewidths=.5, annot=True, cbar_kws={"shrink": .5})

ax.set_xticklabels(

ax.get_xticklabels(),

rotation=45,

horizontalalignment="right"

)

bottom, top = ax.get_ylim()

ax.set_ylim(bottom + 0.5, top - 0.5)

plt.title("Pearson's absolute correlation matrix")

# high correlations between ADH_MIN and ADH_MAX and ADH_MEAN : keep only ADH_MEAN as it has the biggest correlation

df3m = df.drop(["ADH_MIN","ADH_MAX","DOSE_ADHERENCE", "DOSE_ADHERENCE6m",

"DOSE_ADHERENCE9m"], axis=1)

df6m = df.drop(["ADH_MIN","ADH_MAX","DOSE_ADHERENCE", "DOSE_ADHERENCE3m",

"DOSE_ADHERENCE9m"], axis=1)

df9m = df.drop(["ADH_MIN","ADH_MAX","DOSE_ADHERENCE3m", "DOSE_ADHERENCE6m",

"DOSE_ADHERENCE9m"], axis=1)

# re-generate the pearson's abosolute correlation matrix

cat_var = ["GENDER", "INJECTION_SPEED", "INJECTION_DEPTH",

"NEEDLE_SPEED", "HSDS_START_LOW"]

corr_pearson = df.drop(cat_var, axis=1).corr(method="pearson")

# plot correlation matrix

sns.set(style="white")

# Generate a mask for the upper triangle

mask = np.triu(np.ones_like(corr_pearson, dtype=np.bool))

# Set up the matplotlib figure

f, ax = plt.subplots(figsize=(12, 9.5))

# Generate a custom diverging colormap

cmap = sns.diverging_palette(240, 10, n=20)

# Draw the heatmap with the mask and correct aspect ratio

ax = sns.heatmap(corr_pearson.abs(), mask=mask, cmap=cmap, vmin=0.0, vmax=1.0, center=0,

square=True, linewidths=.5, annot=True, cbar_kws={"shrink": .5})

ax.set_xticklabels(

ax.get_xticklabels(),

rotation=45,

horizontalalignment="right"

)

bottom, top = ax.get_ylim()

ax.set_ylim(bottom + 0.5, top - 0.5)

plt.title("Pearson's absolute correlation matrix")

plt.savefig("/plots/Pearsonscorrmonths.png")

# plotting the top 3 features from the first 3 months mostly correlated with adherence in months 4 to 6

toplot = round(corr_pearson.drop("DOSE_ADHERENCE3m",axis=1).loc["DOSE_ADHERENCE3m"],1).loc[["ADH_MEAN", "ADH_STD", "NR_TRANS"]]

sns.set("notebook", style="ticks")

sns.barplot(toplot.values, toplot.index, color=m_darkblue)

plt.xlabel("Pearson's correlation with mean adherence over the 4th to 6th month")

plt.yticks(np.arange(3), ("Mean adherence\nover the first 3 months", "Adherence standard deviation\nover the first 3 months", "Number of transmissions\nover the first 3 months"))

plt.savefig("/plots/top3corr3months.png", dpi=300, bbox_inches="tight")

plt.show()

# plotting the top 3 features from the first 3 months mostly correlated with adherence in months 4 to 9

toplot = round(corr_pearson.drop("DOSE_ADHERENCE6m",axis=1).loc["DOSE_ADHERENCE6m"],1).loc[["ADH_MEAN", "ADH_STD", "NR_TRANS"]]

sns.set("notebook", style="ticks")

sns.barplot(toplot.values, toplot.index, color=m_darkblue)

plt.xlabel("Pearson's correlation with mean adherence over the 4th to 9th month")

plt.yticks(np.arange(3), ("Mean adherence\nover the first 3 months", "Adherence standard deviation\nover the first 3 months", "Number of transmissions\nover the first 3 months"))

plt.savefig("/plots/top3corr6months.png", dpi=300, bbox_inches="tight")

plt.show()

# plotting the top 3 features from the first 3 months mostly correlated with adherence in months 4 to 12

toplot = round(corr_pearson.drop("DOSE_ADHERENCE9m",axis=1).loc["DOSE_ADHERENCE9m"],1).loc[["ADH_MEAN", "ADH_STD", "NR_TRANS"]]

sns.set("notebook", style="ticks")

sns.barplot(toplot.values, toplot.index, color=m_darkblue)

plt.xlabel("Pearson's correlation with mean adherence over the 4th to 12th month")

plt.yticks(np.arange(3), ("Mean adherence\nover the first 3 months", "Adherence standard deviation\nover the first 3 months", "Number of transmissions\nover the first 3 months"))

plt.savefig("/plots/top3corr9months.png", dpi=300, bbox_inches="tight")

plt.show()

#################general characteristics table 1

df.columns

df["AGE_START"].mean()

df["AGE_START"].std()

df["GENDER"].value_counts(normalize=True).round(2)

df["AGE_START"].std()

df["NR_TRANS"].mean()

df["NR_TRANS"].std()

df["NR_TRANS"].quantile([.25, .5,.75])

df["NR_TRANS"].value_counts(normalize=False)

df["INJECTION_TIME"].mean()

df["INJECTION_TIME"].std()

df["INJECTION_TIME"].quantile([.25, .5,.75])

df["ADH_MEAN"].mean()

df["ADH_MEAN"].std()

df["ADH_MEAN"].quantile([.25, .5,.75])

df["NEEDLE_SPEED"].value_counts(normalize=True).round(2)

df["INJECTION_DEPTH"].value_counts(normalize=True).round(2)

df["NR_DOSE_CHANGES"].mean()

df["NR_DOSE_CHANGES"].std()

df["NR_DOSE_CHANGES"].quantile([.25, .5,.75])

df["NR_DOSE_CHANGES"].value_counts(normalize=False)

df["GENDER"].value_counts(normalize=False)

df["NEEDLE_SPEED"].value_counts(normalize=False)

df["INJECTION_DEPTH"].value_counts(normalize=False)

################# future 3 months prediction

#change to 3 months future prediction

df3m = df.drop(["ADH_MIN","ADH_MAX","DOSE_ADHERENCE", "DOSE_ADHERENCE6m",

"DOSE_ADHERENCE9m"], axis=1)

# binning the adherence level : below or above 85% threshold

df3m["ADH_BELOW_THRESH3m"] = pd.cut(df3m["DOSE_ADHERENCE3m"], bins=[0,85,110], include_lowest=True, labels=[1,0])

# creating a feature indicating whether a patient has measurements recorded in the system or not

df3m["HAS_MEAS"] = np.where(df3m[["NR_MEAS", "HSDS_START", "HSDS_START_LOW"]].isnull().any(axis=1), 0, 1)

# get dataset with all patients but without measurement-related features

df3m.set_index("PATIENT_ID", inplace=True)

df3m_all_classif = df3m.drop(["NR_MEAS", "HSDS_START", "HSDS_START_LOW", "DOSE_ADHERENCE3m", "HAS_MEAS"], axis=1)

# get reduced dataset with patients for whom measurement-related features are available

df3m_reduced_classif = df3m[df3m["HAS_MEAS"]==1].drop(["HAS_MEAS", "DOSE_ADHERENCE3m"],axis=1)

print("full dataset shape: ",df3m_all_classif.shape)

print("reduced dataset shape (only patients with measurements):", df3m_reduced_classif.shape)

# what is the class distribution of the target variable? - absolute numbers

df3m_all_classif["ADH_BELOW_THRESH3m"].value_counts(normalize=False)

df3m_all_classif["GENDER"].value_counts(normalize=True).round(2)

df3m_all_classif.groupby("GENDER")["AGE_START"].median()

# looking at class distribution in the whole dataset

dist_full_data = df3m_all_classif["ADH_BELOW_THRESH3m"].value_counts(normalize=True).round(2)

dist_full_data

#0 0.82

#1 0.18

#Name: ADH_BELOW_THRESH3m, dtype: float64

df3m_all_classif["ADH_BELOW_THRESH3m"].value_counts().round(2)

# shuffle the dataset

shuffled_df3m = df3m_all_classif.sample(frac=1,random_state=1)

# put all the "positive" class in a separate dataset (patient below 85% adherence)

below = df3m_all_classif.loc[shuffled_df3m["ADH_BELOW_THRESH3m"] == 1]

# randomly select x observations (the number of "below" classes) from the above medical threshold

above = shuffled_df3m.loc[shuffled_df3m["ADH_BELOW_THRESH3m"] == 0].sample(n=len(below),random_state=1)

# keep the samples non-selected aside (for future testing)

not_sel = shuffled_df3m[(shuffled_df3m["ADH_BELOW_THRESH3m"] == 0) & (~shuffled_df3m.index.isin(set(above.index)))]

# Concatenate "above" and "below" sampled dataframes and shuffle again

undersampled_df3m = pd.concat([below, above]).sample(frac=1, random_state=1)

#plot the dataset after the undersampling

plt.figure(figsize=(8, 8))

sns.countplot("ADH_BELOW_THRESH3m", data=undersampled_df3m)

plt.title("Balanced Classes")

plt.xlabel("Adherence below threshold")

plt.ylabel("Count")

plt.show()

print("undersampled dataframe size: ",undersampled_df3m.shape)

print("remainder dataset of class 0 (above): ", not_sel.shape)

# split data into train (for training and optimizing models in a 5-fold cross-validation scheme) and test set (for assesing model performance)

X = undersampled_df3m.drop("ADH_BELOW_THRESH3m", axis=1)

y = undersampled_df3m["ADH_BELOW_THRESH3m"]

X3m_tr, X_te, Y3m_tr, y_te = train_test_split(X, y, test_size=0.2, random_state=0)

# creating a new test set that follows the 18% - 82% distribution by completing as needed with "above" samples not selected previously

total_needed = int(np.ceil(len(y_te[y_te == 1]) / dist_full_data[1])) #round to the next integer

above_toadd = total_needed - len(y_te)

sample_toadd = not_sel.sample(above_toadd, random_state=0)

X_te_full3m = pd.concat([X_te, sample_toadd.drop("ADH_BELOW_THRESH3m", axis=1)], axis=0)

y_te_full3m = pd.concat([y_te, sample_toadd["ADH_BELOW_THRESH3m"]], axis=0)

print("Training size: ", X3m_tr.shape)

print("matrix Test size: ", X_te_full3m.shape)

print("target test size: ", y_te_full3m.shape)

#undersampled dataframe size: (3928, 11)

#remainder dataset of class 0 (above): (7001, 11)

#Training size: (3142, 10)

#matrix Test size: (2167, 10)

#target test size: (2167,)

# proportion of examples 0 and 1 in the train set

Y3m_tr.value_counts(normalize=True).round(3)*100

Y3m_tr.value_counts()

# proportion of examples 0 and 1 in the test set

y_te_full3m.value_counts(normalize=True).round(3)*100

y_te_full3m.value_counts()

y_te_full3m.shape

# create a dictionary to store optimized algorithms

best_model3m = {}

# score to optimize for

scoring = {

"f1" : "f1" #

}

#random forest

rf = RandomForestClassifier(random_state=1)

# Create cross-validation object

grid_cv = GridSearchCV(rf, {

"n_estimators": [100,300,500,700,900],

"bootstrap": [True]

}, scoring=scoring, cv=5, refit=False)

# Fit estimator

grid_cv.fit(X3m_tr, Y3m_tr)

# Collect results in a DataFrame

df3m_rf = pd.DataFrame.from_dict(dict(

{"n_estimators": grid_cv.cv_results_["param_n_estimators"],

"bootstrap" : grid_cv.cv_results_["param_bootstrap"],

"mean_f1": grid_cv.cv_results_["mean_test_f1"],

"std_f1": grid_cv.cv_results_["std_test_f1"]

}))

df3m_rf.sort_values(by=["mean_f1"], ascending=False).head(5).round(3)

best_model3m["RF_BS_T_300"] = 0.807

#Extra trees

xtc = ExtraTreesClassifier(random_state=1)

grid_cv = GridSearchCV(xtc, {

"n_estimators": [500, 600, 800, 900]

}, scoring=scoring, cv=5, refit=False)

# Fit estimator739

grid_cv.fit(X3m_tr, Y3m_tr)

# Collect results in a DataFrame

df3m_xtc = pd.DataFrame.from_dict(dict(

{"n_estimators": grid_cv.cv_results_["param_n_estimators"],

"mean_f1": grid_cv.cv_results_["mean_test_f1"],

"std_f1": grid_cv.cv_results_["std_test_f1"],

}))

df3m_xtc.sort_values(by=["mean_f1"], ascending=False).head(10).round(3)

best_model3m["ExtraTrees_600"] = 0.801

#Logistic regression

logreg = LogisticRegression(solver="saga", random_state=1, max_iter=5000)

# Create pipeline

pipe = Pipeline([

("scaler", None), # Optional step

("logreg", logreg)

])

# Create cross-validation grid search object

grid_cv = GridSearchCV(pipe, [{

"scaler" : [None, MinMaxScaler(), StandardScaler(), RobustScaler()],

"logreg__penalty" : ["l1", "l2"],

"logreg__C": np.logspace(-2,2,5)

}], scoring=scoring, cv=5, refit=False)

# Fit estimator

grid_cv.fit(X3m_tr, Y3m_tr)

# Collect results in a DataFrame

df3m_logreg = pd.DataFrame.from_dict(dict(

{

"param_scaler" : grid_cv.cv_results_["param_scaler"],

"penalty" : grid_cv.cv_results_["param_logreg__penalty"],

"C" : grid_cv.cv_results_["param_logreg__C"],

"mean_f1": grid_cv.cv_results_["mean_test_f1"],

"std_f1": grid_cv.cv_results_["std_test_f1"],

}))

df3m_logreg.sort_values(by=["mean_f1"], ascending=False).head(5).round(3)

best_model3m["LogReg_RS_l2_C10"] = 0.797

#ordinal logistic regression

olg = mord.LogisticIT()

pipe = Pipeline([

("scaler", None), # Optional step

("olg", olg)

])

# Create cross-validation object

grid_cv = GridSearchCV(pipe, {

"scaler" : [None, StandardScaler(), MinMaxScaler(), RobustScaler()],

"olg__alpha": [.1, 0.3, 1, 3, 10, 30, 100, 300]

}, scoring=scoring, cv=5, refit=False)

# Fit estimator

grid_cv.fit(X3m_tr, Y3m_tr.astype("int"))

# Collect results in a DataFrame

df3m_olr = pd.DataFrame.from_dict(dict(

{

"scaler" : grid_cv.cv_results_["param_scaler"],

"alpha": grid_cv.cv_results_["param_olg__alpha"],

"mean_f1": grid_cv.cv_results_["mean_test_f1"],

"std_f1": grid_cv.cv_results_["std_test_f1"],

}))

df3m_olr.sort_values(by=["mean_f1"], ascending=False).head(5).round(3)

best_model3m["OLR_None_alpha_3"] = 0.798

#optimized algorithms

best_model3m = pd.DataFrame.from_dict(best_model3m, orient="index", columns=["F1"])

best_model3m.sort_values(by=["F1"], ascending=False)

model3m = RandomForestClassifier(n_estimators=300, bootstrap=True, random_state=1)

# plot ROC curves on 5-fold CV on the train set

from sklearn.metrics import auc

from sklearn.metrics import plot_roc_curve

# Classification and ROC analysis

# Run classifier with cross-validation and plot ROC curves

cv = StratifiedKFold(n_splits=5, shuffle=True, random_state=1)

classifier3m = model3m

Xvals = X3m_tr.values

yvals = Y3m_tr.values

tprs = []

aucs = []

mean_fpr = np.linspace(0, 1, 100)

sns.set(style="whitegrid")

fig, ax = plt.subplots(figsize=(8,5))

for i, (train, test) in enumerate(cv.split(Xvals, yvals)):

classifier3m.fit(Xvals[train], yvals[train])

viz = plot_roc_curve(classifier3m, Xvals[test], yvals[test],

name="ROC fold {}".format(i),

alpha=0.3, lw=1, ax=ax)

interp_tpr = np.interp(mean_fpr, viz.fpr, viz.tpr)

interp_tpr[0] = 0.0

tprs.append(interp_tpr)

aucs.append(viz.roc_auc)

ax.plot([0, 1], [0, 1], linestyle="--", lw=2, color="r",

label="Chance", alpha=.8)

mean_tpr = np.mean(tprs, axis=0)

mean_tpr[-1] = 1.0

mean_auc = auc(mean_fpr, mean_tpr)

std_auc = np.std(aucs)

ax.plot(mean_fpr, mean_tpr, color="b",

label=r"Mean ROC (AUC = %0.2f $\pm$ %0.2f)" % (mean_auc, std_auc),

lw=2, alpha=.8)

std_tpr = np.std(tprs, axis=0)

tprs_upper = np.minimum(mean_tpr + std_tpr, 1)

tprs_lower = np.maximum(mean_tpr - std_tpr, 0)

ax.fill_between(mean_fpr, tprs_lower, tprs_upper, color="grey", alpha=.2,

label=r"$\pm$ 1 std. dev.")

ax.set(xlim=[-0.05, 1.05], ylim=[-0.05, 1.05],

title="Receiver operating characteristic - train set, 5 fold cross-validation")

ax.legend(loc="lower right")

plt.show()

# plot ROC curve on test set

from sklearn.metrics import auc

from sklearn.metrics import plot_roc_curve

classifier3m = model3m

classifier3m.fit(X3m_tr, Y3m_tr)

sns.set(style="whitegrid")

fig, ax = plt.subplots(figsize=(8,5))

viz = plot_roc_curve(classifier3m, X_te_full3m, y_te_full3m,

name="ROC", color="b",

alpha=1, lw=2, ax=ax)

ax.plot([0, 1], [0, 1], linestyle="--", lw=2, color="r",

label="Chance", alpha=.8)

ax.set(xlim=[-0.05, 1.05], ylim=[-0.05, 1.05],

title="Receiver operating characteristic - test set (18% - 82% class distribution)")

ax.legend(loc="lower right")

plt.savefig("/plots/roc_curve_test3months.png", dpi=300)

plt.show()

# Metric on the test set - balanced

model3m.fit(X3m_tr, Y3m_tr)

model3m_preds = model3m.predict(X_te_full3m)

tn, fp, fn, tp = confusion_matrix(y_te_full3m, model3m_preds).ravel()

sensitivity = tp / (tp+fn)

specificity = tn / (tn+fp)

print(f"The number of true positives of the model is : {tp:.2f}")

print(f"The number of false negatives of the model is : {fn:.2f}")

print(f"The number of true negatives of the model is : {tn:.2f}")

print(f"The number of false positives of the model is : {fp:.2f}")

print(f"The sensitivity (recall) of the model is : {sensitivity:.2f}")

print(f"The specificity (true negative rate) of the model is : {specificity:.2f}")

print(f"The f1-score of the model is : {metrics.f1_score(y_te_full3m, model3m_preds):.2f}")

#The sensitivity (recall) of the model is : 0.77

#The specificity (true negative rate) of the model is : 0.81

#The f1-score of the model is : 0.59

# confusion matrix on test set - normalized

#fig, ax = plt.subplots(figsize=(8,8))

sns.set(style="white")

plot_confusion_matrix(model3m, X_te_full3m, y_te_full3m, normalize="true")

plt.xticks(np.arange(2), ["Above 85%", "Below 85%"])

plt.yticks(np.arange(2), ["Above 85%", "Below 85%"])

plt.title("Confusion matrix normalized\nRandom Forest model", loc = "left")

plt.savefig("/plots/confusion_matrix_normalized3months.png", dpi=300, bbox_inches="tight")

# plot confusion matrix with absolute numbers

sns.set(style="white")

plot_confusion_matrix(model3m, X_te_full3m, y_te_full3m, values_format='d')

plt.xticks(np.arange(2), ["Above 85%", "Below 85%"])

plt.yticks(np.arange(2), ["Above 85%", "Below 85%"])

plt.title("Confusion matrix\nRandom Forest model", loc = "left")

plt.savefig("/plots/confusion_matrix3months.png", dpi=300, bbox_inches="tight")

#Comparison with a simple baseline model (simple heurestics) based on mean adherence in first months only

# simple model applied only the test set (for 1 to 1 comparison with the ML performances)

comp = X_te_full3m.copy()

comp["Simple_pred"] = np.where(comp["ADH_MEAN"]>85, 0, 1)

y_simple_pred = comp["Simple_pred"]

tn_simple, fp_simple, fn_simple, tp_simple = confusion_matrix(y_te_full3m, y_simple_pred).ravel()

sensitivity_simple = tp_simple / (tp_simple+fn_simple)

specificity_simple = tn_simple / (tn_simple+fp_simple)

print(f"The sensitivity (recall) of the simple heuristics model is : {sensitivity_simple:.2f}")

print(f"The specificity (true negative rate) of the simple heuristics model is : {specificity_simple:.2f}")

print(f"The f1-score of the simple heuristics model is : {metrics.f1_score(y_te_full3m, y_simple_pred):.2f}")

#The sensitivity (recall) of the simple heuristics model is : 0.49

#The specificity (true negative rate) of the simple heuristics model is : 0.96

#The f1-score of the simple heuristics model is : 0.60

cm = confusion_matrix(y_te_full3m, y_simple_pred, normalize="true")

plt.figure(figsize=(5,4))

g = sns.heatmap(

cm,

cmap="viridis",

vmin=0.2, vmax=0.8,

annot=True,

xticklabels=["Above 85%", "Below 85%"],

yticklabels=["Above 85%", "Below 85%"])

g.set_yticklabels(g.get_yticklabels(), rotation = 0)

plt.xlabel("Predicted label")

plt.ylabel("True label")

plt.title("Confusion matrix normalized\nsimple heuristic mode", loc = "left")

plt.savefig("/plots/confusion_matrix_normalized_simple_heuristics3months.png", dpi=300, bbox_inches="tight")

cm = confusion_matrix(y_te_full3m, y_simple_pred)

plt.figure(figsize=(5,4))

g = sns.heatmap(

cm,

cmap="viridis",

vmin=100, vmax=1500,

annot=True,

fmt="d",

xticklabels=["Above 85%", "Below 85%"],

yticklabels=["Above 85%", "Below 85%"])

g.set_yticklabels(g.get_yticklabels(), rotation = 0)

plt.xlabel("Predicted label")

plt.ylabel("True label")

plt.title("Confusion matrix normalized\nsimple heuristic model", loc = "left")

plt.savefig("/plots/confusion_matrix_simple_heuristics3months.png", dpi=300, bbox_inches="tight")

# comparing f1-scores

f1_ml = metrics.f1_score(y_te_full3m, model3m_preds)

f1_simple = metrics.f1_score(y_te_full3m, y_simple_pred)

pd.DataFrame(data=[f1_ml, f1_simple], columns=["F1 score"], index=["ML Model", "Simple Model"]).round(3)

# F1 score

#ML Model 0.586

#Simple Model 0.596

# plot ROC curve on test set (2 plots)

from sklearn.metrics import auc

from sklearn.metrics import plot_roc_curve

classifier3m = model3m

onlyadh3m = classifier3m.fit(

X3m_tr[["ADH_MEAN", "ADH_STD"]] #.values.reshape(-1,1)

,Y3m_tr

)

sns.set(style="whitegrid")

fig, ax = plt.subplots()

viz3m = plot_roc_curve(onlyadh3m, X_te_full3m[["ADH_MEAN", "ADH_STD"]] #.values.reshape(-1,1)

, y_te_full3m,

name="ROC Adherence only", color=m_darkblue,

alpha=1, lw=2, ax=ax)

allfeat3m = classifier3m.fit(X3m_tr, Y3m_tr)

viz23m = plot_roc_curve(allfeat3m, X_te_full3m, y_te_full3m,

name="ROC All features", color=m_darkgreen,

alpha=1, lw=2, ax=ax)

ax.plot([0, 1], [0, 1], linestyle="--", lw=2, color="r",

label="Chance", alpha=.8)

ax.set(xlim=[-0.05, 1.05], ylim=[-0.05, 1.05],

title="Receiver operating characteristic - test set")

ax.legend(loc="lower right")

plt.savefig("/plots/roc_curve_comp_onlyadh3months.png", dpi=300)

plt.show()

#Machine learning model interpretation

explainer = shap.TreeExplainer(model3m)

shap_values = explainer.shap_values(X_te_full3m)

# feature importance plot; importance is ordered by descreasing

shap.summary_plot(shap_values[1], X_te_full3m, plot_type="bar")

shap.summary_plot(shap_values[1], X_te_full3m, show=False)

plt.savefig("/plots/shap_plot3months.png", dpi=300, bbox_inches="tight")

#interpretation with LIME

# rename columns (shorter names needed)

X3m_tr_renamed = X3m_tr.copy().rename({

"AGE_START":"age", # age at start of use

"START_MONTH":"mstart", # month at start of use

"ADH_MEAN":"adh_mean", # mean adherence over the first 3 months

"ADH_STD":"adh_std", # adherence standard deviation over the first 3 months

"NR_TRANS":"nr_trans", # nr of transmissions in the first 3 months

"INJECTION_TIME":"itime", # injection time setting (s)

"INJECTION_DEPTH":"idepth", # injection depth setting (mm)

"INJECTION_SPEED" : "ispeed", # injection speed setting (0:slow, 1:medium, 2:fast)

"NEEDLE_SPEED":"nspeed", # needle speed setting (0:slow, 1:medium, 2:fast)

"NR_DOSE_CHANGES": "dosech", # number of dose changes in the first 3 months

"GENDER" : "gender" # gender (0: girl, 1:boy)

}, axis=1)

#random sampling 10 instance where the real adherence to be predicted should be "below", respectively "above"

below_explain = y_te_full3m[y_te_full3m == 1].sample(10, random_state=1).index.tolist()

above_explain = y_te_full3m[y_te_full3m == 0].sample(10, random_state=1).index.tolist()

# set up and fit lime model explainer

predict_model3m = lambda x: model3m.predict_proba(x).astype(float)

explainer_model3m = lime.lime_tabular.LimeTabularExplainer(

X3m_tr.values,

feature_names = X3m_tr_renamed.columns,

categorical_features=["boy"],

class_names=["Above","Below"],

kernel_width=5

)

# explaining class "below 85%"

for instance in below_explain:

print("real class: below 85% adherence")

choosen_instance = X_te_full3m.loc[[instance]].values[0]

display(X_te_full3m.loc[[instance]])

print("interpretation")

exp_model3m = explainer_model3m.explain_instance(choosen_instance, predict_model3m, num_features=11)

exp_model3m.show_in_notebook(show_all=True)

exp_model3m.save_to_file(f"instance_below_{instance}.html")

# explaining class 0

for instance in above_explain:

choosen_instance = X_te_full3m.loc[[instance]].values[0]

display(X_te_full3m.loc[[instance]])

print("interpretation")

exp_model3m = explainer_model3m.explain_instance(choosen_instance, predict_model3m, num_features=11)

exp_model3m.show_in_notebook(show_all=True)

exp_model3m.save_to_file(f"instance_above_{instance}.html")

# make a copy of the dataframe for hypothesis testing purposes

df3m_hypothesis = undersampled_df3m.copy()

# bin features in categories as defined above

df3m_hypothesis["ADH_BELOW_THRESH3m"] = np.where(df3m_hypothesis["ADH_BELOW_THRESH3m"]==1, "below thresh.", "above thresh.")

df3m_hypothesis["AGE_START"] = np.where(df3m_hypothesis["AGE_START"]>10, ">10", "<=10")

df3m_hypothesis["GENDER"] = np.where(df3m_hypothesis["GENDER"]==1, "Boy", "Girl")

df3m_hypothesis["NR_TRANS"] = np.where(df3m_hypothesis["NR_TRANS"]==0, "0", ">0")

df3m_hypothesis["ADH_MEAN"] = np.where(df3m_hypothesis["ADH_MEAN"]< 90, "<90", ">=90")

df3m_hypothesis["ADH_STD"] = np.where(df3m_hypothesis["ADH_STD"]>9, ">9", "<=9")

df3m_hypothesis["NEEDLE_SPEED"] = np.where(df3m_hypothesis["NEEDLE_SPEED"]<=1, "low/medium", "fast")

df3m_hypothesis["INJECTION_TIME"] = np.where(df3m_hypothesis["INJECTION_TIME"]<=5, "<=5", ">5")

df3m_hypothesis["NR_DOSE_CHANGES"] = np.where(df3m_hypothesis["NR_DOSE_CHANGES"] == 0, "0", ">0")

df3m_hypothesis["INJECTION_DEPTH"] = np.where(df3m_hypothesis["INJECTION_DEPTH"]>6, "<=6", ">6")

# perform chi2 statistical tests for each of the features binned previously

stats_p_values = {}

for column in df3m_hypothesis.drop(["ADH_BELOW_THRESH3m", "INJECTION_SPEED"], axis=1).columns:

ct = pd.crosstab(df3m_hypothesis[column], df3m_hypothesis["ADH_BELOW_THRESH3m"])

chi2, p, dof, ex = chi2_contingency(observed=ct)

stats_p_values[column] = [chi2, p]

# gather test results (feature and associated p-value in a dataframe)

df3m_tests = pd.DataFrame.from_dict(stats_p_values, orient="index", columns=["Chi2", "p_value"]).reset_index().sort_values(by="p_value", ascending=True)

df3m_tests = df3m_tests.rename({"index":"Feature"}, axis=1)

# display all results ordered by p-value in ascending order

display(df3m_tests.drop("Chi2", axis=1).round(3))

# only display results for which the p-value is below 0.05 significance threshold

display(df3m_tests[df3m_tests["p_value"]<0.05].drop("Chi2", axis=1))

# recalculate the bonferroni corrected p-value

# (accounting for the fact that sample size may differ and that multiple tests are performed)

reject, p_adjusted, _, _ = multipletests(df3m_tests["p_value"], method="bonferroni")

# gather bonferroni corrected p-values in a dataframe

df3m_tests_bc = (

pd.DataFrame(

list(zip(df3m_tests["Feature"].values, p_adjusted, reject)),

columns=["Feature", "bf-corrected p_value", "Reject"]

)

.sort_values(by="bf-corrected p_value", ascending=True).round(3)

)

df3m_tests_bc = df3m_tests_bc.rename({"index":"Feature"}, axis=1)

# display only statistically significant features under a bonferroni corrected p-value

display(df3m_tests_bc[df3m_tests_bc["Reject"] == True])

################# future 6 months prediction

#change to 6 months future prediction

df6m = df.drop(["ADH_MIN","ADH_MAX","DOSE_ADHERENCE", "DOSE_ADHERENCE3m",

"DOSE_ADHERENCE9m"], axis=1)

# binning the adherence level : below or above 85% threshold

df6m["ADH_BELOW_THRESH6m"] = pd.cut(df6m["DOSE_ADHERENCE6m"], bins=[0,85,110], include_lowest=True, labels=[1,0])

# creating a feature indicating whether a patient has measurements recorded in the system or not

df6m["HAS_MEAS"] = np.where(df6m[["NR_MEAS", "HSDS_START", "HSDS_START_LOW"]].isnull().any(axis=1), 0, 1)

# get dataset with all patients but without measurement-related features

df6m.set_index("PATIENT_ID", inplace=True)

df6m_all_classif = df6m.drop(["NR_MEAS", "HSDS_START", "HSDS_START_LOW", "DOSE_ADHERENCE6m", "HAS_MEAS"], axis=1)

# get reduced dataset with patients for whom measurement-related features are available

df6m_reduced_classif = df6m[df6m["HAS_MEAS"]==1].drop(["HAS_MEAS", "DOSE_ADHERENCE6m"],axis=1)

print("full dataset shape: ",df6m_all_classif.shape)

print("reduced dataset shape (only patients with measurements):", df6m_reduced_classif.shape)

# what is the class distribution of the target variable? - absolute numbers

df6m_all_classif["ADH_BELOW_THRESH6m"].value_counts(normalize=False)

df6m_all_classif["GENDER"].value_counts(normalize=True).round(2)

df6m_all_classif.groupby("GENDER")["AGE_START"].median()

# looking at class distribution in the whole dataset

dist_full_data = df6m_all_classif["ADH_BELOW_THRESH6m"].value_counts(normalize=True).round(2)

dist_full_data

#0 0.8

#1 0.2

#Name: ADH_BELOW_THRESH6m, dtype: float64

df6m_all_classif["ADH_BELOW_THRESH6m"].value_counts().round(2)

# shuffle the dataset

shuffled_df6m = df6m_all_classif.sample(frac=1,random_state=1)

# put all the "positive" class in a separate dataset (patient below 85% adherence)

below = df6m_all_classif.loc[shuffled_df6m["ADH_BELOW_THRESH6m"] == 1]

# randomly select x observations (the number of "below" classes) from the above medical threshold

above = shuffled_df6m.loc[shuffled_df6m["ADH_BELOW_THRESH6m"] == 0].sample(n=len(below),random_state=1)

# keep the samples non-selected aside (for future testing)

not_sel = shuffled_df6m[(shuffled_df6m["ADH_BELOW_THRESH6m"] == 0) & (~shuffled_df6m.index.isin(set(above.index)))]

# Concatenate "above" and "below" sampled dataframes and shuffle again

undersampled_df6m = pd.concat([below, above]).sample(frac=1, random_state=1)

#plot the dataset after the undersampling

plt.figure(figsize=(8, 8))

sns.countplot("ADH_BELOW_THRESH6m", data=undersampled_df6m)

plt.title("Balanced Classes")

plt.xlabel("Adherence below threshold")

plt.ylabel("Count")

plt.show()

print("undersampled dataframe size: ",undersampled_df6m.shape)

print("remainder dataset of class 0 (above): ", not_sel.shape)

# split data into train (for training and optimizing models in a 5-fold cross-validation scheme) and test set (for assesing model performance)

X = undersampled_df6m.drop("ADH_BELOW_THRESH6m", axis=1)

y = undersampled_df6m["ADH_BELOW_THRESH6m"]

X6m_tr, X_te, Y6m_tr, y_te = train_test_split(X, y, test_size=0.2, random_state=0)

# creating a new test set that follows the 20% - 80% distribution by completing as needed with "above" samples not selected previously

total_needed = int(np.ceil(len(y_te[y_te == 1]) / dist_full_data[1])) #round to the next integer

above_toadd = total_needed - len(y_te)

sample_toadd = not_sel.sample(above_toadd, random_state=0)

X_te_full6m = pd.concat([X_te, sample_toadd.drop("ADH_BELOW_THRESH6m", axis=1)], axis=0)

y_te_full6m = pd.concat([y_te, sample_toadd["ADH_BELOW_THRESH6m"]], axis=0)

print("Training size: ", X6m_tr.shape)

print("matrix Test size: ", X_te_full6m.shape)

print("target test size: ", y_te_full6m.shape)

#undersampled dataframe size: (4388, 11)

#remainder dataset of class 0 (above): (6541, 11)

#Training size: (3510, 10)

#matrix Test size: (2045, 10)

#target test size: (2045,)

# proportion of examples 0 and 1 in the train set

Y6m_tr.value_counts(normalize=True).round(3)*100

Y6m_tr.value_counts()

# proportion of examples 0 and 1 in the test set

y_te_full6m.value_counts(normalize=True).round(3)*100

y_te_full6m.value_counts()

y_te_full6m.shape

# create a dictionary to store optimized algorithms

best_model6m = {}

# score to optimize for

scoring = {

"f1" : "f1" #

}

#random forest

rf = RandomForestClassifier(random_state=1)

# Create cross-validation object

grid_cv = GridSearchCV(rf, {

"n_estimators": [100,300,500,700,900],

"bootstrap": [True]

}, scoring=scoring, cv=5, refit=False)

# Fit estimator

grid_cv.fit(X6m_tr, Y6m_tr)

# Collect results in a DataFrame

df6m_rf = pd.DataFrame.from_dict(dict(

{"n_estimators": grid_cv.cv_results_["param_n_estimators"],

"bootstrap" : grid_cv.cv_results_["param_bootstrap"],

"mean_f1": grid_cv.cv_results_["mean_test_f1"],

"std_f1": grid_cv.cv_results_["std_test_f1"]

}))

df6m_rf.sort_values(by=["mean_f1"], ascending=False).head(5).round(3)

best_model6m["RF_BS_T_300"] = 0.798

#Extra trees

xtc = ExtraTreesClassifier(random_state=1)

grid_cv = GridSearchCV(xtc, {

"n_estimators": [500, 600, 800, 900]

}, scoring=scoring, cv=5, refit=False)

# Fit estimator739

grid_cv.fit(X6m_tr, Y6m_tr)

# Collect results in a DataFrame

df6m_xtc = pd.DataFrame.from_dict(dict(

{"n_estimators": grid_cv.cv_results_["param_n_estimators"],

"mean_f1": grid_cv.cv_results_["mean_test_f1"],

"std_f1": grid_cv.cv_results_["std_test_f1"],

}))

df6m_xtc.sort_values(by=["mean_f1"], ascending=False).head(10).round(3)

best_model6m["ExtraTrees_500"] = 0.783

#Logistic regression

logreg = LogisticRegression(solver="saga", random_state=1, max_iter=5000)

# Create pipeline

pipe = Pipeline([

("scaler", None), # Optional step

("logreg", logreg)

])

# Create cross-validation grid search object

grid_cv = GridSearchCV(pipe, [{

"scaler" : [None, MinMaxScaler(), StandardScaler(), RobustScaler()],

"logreg__penalty" : ["l1", "l2"],

"logreg__C": np.logspace(-2,2,5)

}], scoring=scoring, cv=5, refit=False)

# Fit estimator

grid_cv.fit(X6m_tr, Y6m_tr)

# Collect results in a DataFrame

df6m_logreg = pd.DataFrame.from_dict(dict(

{

"param_scaler" : grid_cv.cv_results_["param_scaler"],

"penalty" : grid_cv.cv_results_["param_logreg__penalty"],

"C" : grid_cv.cv_results_["param_logreg__C"],

"mean_f1": grid_cv.cv_results_["mean_test_f1"],

"std_f1": grid_cv.cv_results_["std_test_f1"],

}))

df6m_logreg.sort_values(by=["mean_f1"], ascending=False).head(5).round(3)

best_model6m["LogReg_RS_l2_C100"] = 0.785

#ordinal logistic regression

olg = mord.LogisticIT()

pipe = Pipeline([

("scaler", None), # Optional step

("olg", olg)

])

# Create cross-validation object

grid_cv = GridSearchCV(pipe, {

"scaler" : [None, StandardScaler(), MinMaxScaler(), RobustScaler()],

"olg__alpha": [.1, 0.3, 1, 3, 10, 30, 100, 300]

}, scoring=scoring, cv=5, refit=False)

# Fit estimator

grid_cv.fit(X6m_tr, Y6m_tr.astype("int"))

# Collect results in a DataFrame

df6m_olr = pd.DataFrame.from_dict(dict(

{

"scaler" : grid_cv.cv_results_["param_scaler"],

"alpha": grid_cv.cv_results_["param_olg__alpha"],

"mean_f1": grid_cv.cv_results_["mean_test_f1"],

"std_f1": grid_cv.cv_results_["std_test_f1"],

}))

df6m_olr.sort_values(by=["mean_f1"], ascending=False).head(5).round(3)

best_model6m["OLR_None_alpha_10"] = 0.784

#optimized algorithms

best_model6m = pd.DataFrame.from_dict(best_model6m, orient="index", columns=["F1"])

best_model6m.sort_values(by=["F1"], ascending=False)

model6m = RandomForestClassifier(n_estimators=300, bootstrap=True, random_state=1)

# plot ROC curves on 5-fold CV on the train set

from sklearn.metrics import auc

from sklearn.metrics import plot_roc_curve

# Classification and ROC analysis

# Run classifier with cross-validation and plot ROC curves

cv = StratifiedKFold(n_splits=5, shuffle=True, random_state=1)

classifier6m = model6m

Xvals = X6m_tr.values

yvals = Y6m_tr.values

tprs = []

aucs = []

mean_fpr = np.linspace(0, 1, 100)

sns.set(style="whitegrid")

fig, ax = plt.subplots(figsize=(8,5))

for i, (train, test) in enumerate(cv.split(Xvals, yvals)):

classifier6m.fit(Xvals[train], yvals[train])

viz = plot_roc_curve(classifier6m, Xvals[test], yvals[test],

name="ROC fold {}".format(i),

alpha=0.3, lw=1, ax=ax)

interp_tpr = np.interp(mean_fpr, viz.fpr, viz.tpr)

interp_tpr[0] = 0.0

tprs.append(interp_tpr)

aucs.append(viz.roc_auc)

ax.plot([0, 1], [0, 1], linestyle="--", lw=2, color="r",

label="Chance", alpha=.8)

mean_tpr = np.mean(tprs, axis=0)

mean_tpr[-1] = 1.0

mean_auc = auc(mean_fpr, mean_tpr)

std_auc = np.std(aucs)

ax.plot(mean_fpr, mean_tpr, color="b",

label=r"Mean ROC (AUC = %0.2f $\pm$ %0.2f)" % (mean_auc, std_auc),

lw=2, alpha=.8)

std_tpr = np.std(tprs, axis=0)

tprs_upper = np.minimum(mean_tpr + std_tpr, 1)

tprs_lower = np.maximum(mean_tpr - std_tpr, 0)

ax.fill_between(mean_fpr, tprs_lower, tprs_upper, color="grey", alpha=.2,

label=r"$\pm$ 1 std. dev.")

ax.set(xlim=[-0.05, 1.05], ylim=[-0.05, 1.05],

title="Receiver operating characteristic - train set, 5 fold cross-validation")

ax.legend(loc="lower right")

plt.show()

# plot ROC curve on test set

from sklearn.metrics import auc

from sklearn.metrics import plot_roc_curve

classifier6m = model6m

classifier6m.fit(X6m_tr, Y6m_tr)

sns.set(style="whitegrid")

fig, ax = plt.subplots(figsize=(8,5))

viz = plot_roc_curve(classifier6m, X_te_full6m, y_te_full6m,

name="ROC", color="b",

alpha=1, lw=2, ax=ax)

ax.plot([0, 1], [0, 1], linestyle="--", lw=2, color="r",

label="Chance", alpha=.8)

ax.set(xlim=[-0.05, 1.05], ylim=[-0.05, 1.05],

title="Receiver operating characteristic - test set (20% - 80% class distribution)")

ax.legend(loc="lower right")

plt.savefig("/plots/roc_curve_test6months.png", dpi=300)

plt.show()

# Metric on the test set - balanced

model6m.fit(X6m_tr, Y6m_tr)

model6m_preds = model6m.predict(X_te_full6m)

tn, fp, fn, tp = confusion_matrix(y_te_full6m, model6m_preds).ravel()

sensitivity = tp / (tp+fn)

specificity = tn / (tn+fp)

print(f"The sensitivity (recall) of the model is : {sensitivity:.2f}")

print(f"The specificity (true negative rate) of the model is : {specificity:.2f}")

print(f"The f1-score of the model is : {metrics.f1_score(y_te_full6m, model6m_preds):.2f}")

#The sensitivity (recall) of the model is : 0.77

#The specificity (true negative rate) of the model is : 0.81

#The f1-score of the model is : 0.59

# confusion matrix on test set - normalized

#fig, ax = plt.subplots(figsize=(8,8))

sns.set(style="white")

plot_confusion_matrix(model6m, X_te_full6m, y_te_full6m, normalize="true")

plt.xticks(np.arange(2), ["Above 85%", "Below 85%"])

plt.yticks(np.arange(2), ["Above 85%", "Below 85%"])

plt.title("Confusion matrix normalized\nRandom Forest model", loc = "left")

plt.savefig("/plots/confusion_matrix_normalized6months.png", dpi=300, bbox_inches="tight")

# plot confusion matrix with absolute numbers

sns.set(style="white")

plot_confusion_matrix(model6m, X_te_full6m, y_te_full6m, values_format='d')

plt.xticks(np.arange(2), ["Above 85%", "Below 85%"])

plt.yticks(np.arange(2), ["Above 85%", "Below 85%"])

plt.title("Confusion matrix\nRandom Forest model", loc = "left")

plt.savefig("/plots/confusion_matrix6months.png", dpi=300, bbox_inches="tight")

#Comparison with a simple baseline model (simple heurestics) based on mean adherence in first months only

# simple model applied only the test set (for 1 to 1 comparison with the ML performances)

comp = X_te_full6m.copy()

comp["Simple_pred"] = np.where(comp["ADH_MEAN"]>85, 0, 1)

y_simple_pred = comp["Simple_pred"]

tn_simple, fp_simple, fn_simple, tp_simple = confusion_matrix(y_te_full6m, y_simple_pred).ravel()

sensitivity_simple = tp_simple / (tp_simple+fn_simple)

specificity_simple = tn_simple / (tn_simple+fp_simple)

print(f"The sensitivity (recall) of the simple heuristics model is : {sensitivity_simple:.2f}")

print(f"The specificity (true negative rate) of the simple heuristics model is : {specificity_simple:.2f}")

print(f"The f1-score of the simple heuristics model is : {metrics.f1_score(y_te_full6m, y_simple_pred):.2f}")

#The sensitivity (recall) of the simple heuristics model is : 0.44

#The specificity (true negative rate) of the simple heuristics model is : 0.97

#The f1-score of the simple heuristics model is : 0.57

cm = confusion_matrix(y_te_full6m, y_simple_pred, normalize="true")

plt.figure(figsize=(5,4))

g = sns.heatmap(

cm,

cmap="viridis",

vmin=0.2, vmax=0.8,

annot=True,

xticklabels=["Above 85%", "Below 85%"],

yticklabels=["Above 85%", "Below 85%"])

g.set_yticklabels(g.get_yticklabels(), rotation = 0)

plt.xlabel("Predicted label")

plt.ylabel("True label")

plt.title("Confusion matrix normalized\nsimple heuristic mode", loc = "left")

plt.savefig("/plots/confusion_matrix_normalized_simple_heuristics6months.png", dpi=300, bbox_inches="tight")

cm = confusion_matrix(y_te_full6m, y_simple_pred)

plt.figure(figsize=(5,4))

g = sns.heatmap(

cm,

cmap="viridis",

vmin=100, vmax=1500,

annot=True,

fmt="d",

xticklabels=["Above 85%", "Below 85%"],

yticklabels=["Above 85%", "Below 85%"])

g.set_yticklabels(g.get_yticklabels(), rotation = 0)

plt.xlabel("Predicted label")

plt.ylabel("True label")

plt.title("Confusion matrix normalized\nsimple heuristic model", loc = "left")

plt.savefig("/plots/confusion_matrix_simple_heuristics6months.png", dpi=300, bbox_inches="tight")

# comparing f1-scores

f1_ml = metrics.f1_score(y_te_full6m, model6m_preds)

f1_simple = metrics.f1_score(y_te_full6m, y_simple_pred)

pd.DataFrame(data=[f1_ml, f1_simple], columns=["F1 score"], index=["ML Model", "Simple Model"]).round(3)

# F1 score

#ML Model 0.586

#Simple Model 0.596

# plot ROC curve on test set (2 plots)

from sklearn.metrics import auc

from sklearn.metrics import plot_roc_curve

classifier6m = model6m

onlyadh6m = classifier6m.fit(

X6m_tr[["ADH_MEAN", "ADH_STD"]] #.values.reshape(-1,1)

,Y6m_tr

)

sns.set(style="whitegrid")

fig, ax = plt.subplots()

viz6m = plot_roc_curve(onlyadh6m, X_te_full6m[["ADH_MEAN", "ADH_STD"]] #.values.reshape(-1,1)

, y_te_full6m,

name="ROC Adherence only", color=m_darkblue,

alpha=1, lw=2, ax=ax)

allfeat6m = classifier6m.fit(X6m_tr, Y6m_tr)

viz26m = plot_roc_curve(allfeat6m, X_te_full6m, y_te_full6m,

name="ROC All features", color=m_darkgreen,

alpha=1, lw=2, ax=ax)

ax.plot([0, 1], [0, 1], linestyle="--", lw=2, color="r",

label="Chance", alpha=.8)

ax.set(xlim=[-0.05, 1.05], ylim=[-0.05, 1.05],

title="Receiver operating characteristic - test set")

ax.legend(loc="lower right")

plt.savefig("/plots/roc_curve_comp_onlyadh6months.png", dpi=300)

plt.show()

#Machine learning model interpretation

explainer = shap.TreeExplainer(model6m)

shap_values = explainer.shap_values(X_te_full6m)

# feature importance plot; importance is ordered by descreasing

shap.summary_plot(shap_values[1], X_te_full6m, plot_type="bar")

shap.summary_plot(shap_values[1], X_te_full6m, show=False)

plt.savefig("/plots/shap_plot6months.png", dpi=300, bbox_inches="tight")

#interpretation with LIME

# rename columns (shorter names needed)

X6m_tr_renamed = X6m_tr.copy().rename({

"AGE_START":"age", # age at start of use

"START_MONTH":"mstart", # month at start of use

"ADH_MEAN":"adh_mean", # mean adherence over the first 3 months

"ADH_STD":"adh_std", # adherence standard deviation over the first 3 months

"NR_TRANS":"nr_trans", # nr of transmissions in the first 3 months

"INJECTION_TIME":"itime", # injection time setting (s)

"INJECTION_DEPTH":"idepth", # injection depth setting (mm)

"INJECTION_SPEED" : "ispeed", # injection speed setting (0:slow, 1:medium, 2:fast)

"NEEDLE_SPEED":"nspeed", # needle speed setting (0:slow, 1:medium, 2:fast)

"NR_DOSE_CHANGES": "dosech", # number of dose changes in the first 3 months

"GENDER" : "gender" # gender (0: girl, 1:boy)

}, axis=1)

#random sampling 10 instance where the real adherence to be predicted should be "below", respectively "above"

below_explain = y_te_full6m[y_te_full6m == 1].sample(10, random_state=1).index.tolist()

above_explain = y_te_full6m[y_te_full6m == 0].sample(10, random_state=1).index.tolist()

# set up and fit lime model explainer

predict_model6m = lambda x: model6m.predict_proba(x).astype(float)

explainer_model6m = lime.lime_tabular.LimeTabularExplainer(

X6m_tr.values,

feature_names = X6m_tr_renamed.columns,

categorical_features=["boy"],

class_names=["Above","Below"],

kernel_width=5

)

# explaining class "below 85%"

for instance in below_explain:

print("real class: below 85% adherence")

choosen_instance = X_te_full6m.loc[[instance]].values[0]

display(X_te_full6m.loc[[instance]])

print("interpretation")

exp_model6m = explainer_model6m.explain_instance(choosen_instance, predict_model6m, num_features=11)

exp_model6m.show_in_notebook(show_all=True)

exp_model6m.save_to_file(f"instance_below_{instance}.html")

# explaining class 0

for instance in above_explain:

choosen_instance = X_te_full6m.loc[[instance]].values[0]

display(X_te_full6m.loc[[instance]])

print("interpretation")

exp_model6m = explainer_model6m.explain_instance(choosen_instance, predict_model6m, num_features=11)

exp_model6m.show_in_notebook(show_all=True)

exp_model6m.save_to_file(f"instance_above_{instance}.html")

# make a copy of the dataframe for hypothesis testing purposes

df6m_hypothesis = undersampled_df6m.copy()

# bin features in categories as defined above

df6m_hypothesis["ADH_BELOW_THRESH6m"] = np.where(df6m_hypothesis["ADH_BELOW_THRESH6m"]==1, "below thresh.", "above thresh.")

df6m_hypothesis["AGE_START"] = np.where(df6m_hypothesis["AGE_START"]>10, ">10", "<=10")

df6m_hypothesis["GENDER"] = np.where(df6m_hypothesis["GENDER"]==1, "Boy", "Girl")

df6m_hypothesis["NR_TRANS"] = np.where(df6m_hypothesis["NR_TRANS"]==0, "0", ">0")

df6m_hypothesis["ADH_MEAN"] = np.where(df6m_hypothesis["ADH_MEAN"]< 90, "<90", ">=90")

df6m_hypothesis["ADH_STD"] = np.where(df6m_hypothesis["ADH_STD"]>9, ">9", "<=9")

df6m_hypothesis["NEEDLE_SPEED"] = np.where(df6m_hypothesis["NEEDLE_SPEED"]<=1, "low/medium", "fast")

df6m_hypothesis["INJECTION_TIME"] = np.where(df6m_hypothesis["INJECTION_TIME"]<=5, "<=5", ">5")

df6m_hypothesis["NR_DOSE_CHANGES"] = np.where(df6m_hypothesis["NR_DOSE_CHANGES"] == 0, "0", ">0")

df6m_hypothesis["INJECTION_DEPTH"] = np.where(df6m_hypothesis["INJECTION_DEPTH"]>6, "<=6", ">6")

# perform chi2 statistical tests for each of the features binned previously

stats_p_values = {}

for column in df6m_hypothesis.drop(["ADH_BELOW_THRESH6m", "INJECTION_SPEED"], axis=1).columns:

ct = pd.crosstab(df6m_hypothesis[column], df6m_hypothesis["ADH_BELOW_THRESH6m"])

chi2, p, dof, ex = chi2_contingency(observed=ct)

stats_p_values[column] = [chi2, p]

# gather test results (feature and associated p-value in a dataframe)

df6m_tests = pd.DataFrame.from_dict(stats_p_values, orient="index", columns=["Chi2", "p_value"]).reset_index().sort_values(by="p_value", ascending=True)

df6m_tests = df6m_tests.rename({"index":"Feature"}, axis=1)

# display all results ordered by p-value in ascending order

display(df6m_tests.drop("Chi2", axis=1).round(3))

# only display results for which the p-value is below 0.05 significance threshold

display(df6m_tests[df6m_tests["p_value"]<0.05].drop("Chi2", axis=1))

# recalculate the bonferroni corrected p-value

# (accounting for the fact that sample size may differ and that multiple tests are performed)

reject, p_adjusted, _, _ = multipletests(df6m_tests["p_value"], method="bonferroni")

# gather bonferroni corrected p-values in a dataframe

df6m_tests_bc = (

pd.DataFrame(

list(zip(df6m_tests["Feature"].values, p_adjusted, reject)),

columns=["Feature", "bf-corrected p_value", "Reject"]

)

.sort_values(by="bf-corrected p_value", ascending=True).round(3)

)

df6m_tests_bc = df6m_tests_bc.rename({"index":"Feature"}, axis=1)

# display only statistically significant features under a bonferroni corrected p-value

display(df6m_tests_bc[df6m_tests_bc["Reject"] == True])

#####################9 months prediction

################# future 9 months prediction

#change to 9 months future prediction

df9m = df.drop(["ADH_MIN","ADH_MAX","DOSE_ADHERENCE", "DOSE_ADHERENCE3m",

"DOSE_ADHERENCE6m"], axis=1)

# binning the adherence level : below or above 85% threshold

df9m["ADH_BELOW_THRESH9m"] = pd.cut(df9m["DOSE_ADHERENCE9m"], bins=[0,85,110], include_lowest=True, labels=[1,0])

# creating a feature indicating whether a patient has measurements recorded in the system or not

df9m["HAS_MEAS"] = np.where(df9m[["NR_MEAS", "HSDS_START", "HSDS_START_LOW"]].isnull().any(axis=1), 0, 1)

# get dataset with all patients but without measurement-related features

df9m.set_index("PATIENT_ID", inplace=True)

df9m_all_classif = df9m.drop(["NR_MEAS", "HSDS_START", "HSDS_START_LOW", "DOSE_ADHERENCE9m", "HAS_MEAS"], axis=1)

# get reduced dataset with patients for whom measurement-related features are available

df9m_reduced_classif = df9m[df9m["HAS_MEAS"]==1].drop(["HAS_MEAS", "DOSE_ADHERENCE9m"],axis=1)

print("full dataset shape: ",df9m_all_classif.shape)

print("reduced dataset shape (only patients with measurements):", df9m_reduced_classif.shape)

# what is the class distribution of the target variable? - absolute numbers

df9m_all_classif["ADH_BELOW_THRESH9m"].value_counts(normalize=False)

df9m_all_classif["GENDER"].value_counts(normalize=True).round(2)

df9m_all_classif.groupby("GENDER")["AGE_START"].median()

# looking at class distribution in the whole dataset

dist_full_data = df9m_all_classif["ADH_BELOW_THRESH9m"].value_counts(normalize=True).round(2)

dist_full_data

#0 0.78

#1 0.22

#Name: ADH_BELOW_THRESH9m, dtype: float64

df9m_all_classif["ADH_BELOW_THRESH9m"].value_counts().round(2)

# shuffle the dataset

shuffled_df9m = df9m_all_classif.sample(frac=1,random_state=1)

# put all the "positive" class in a separate dataset (patient below 85% adherence)

below = df9m_all_classif.loc[shuffled_df9m["ADH_BELOW_THRESH9m"] == 1]

# randomly select x observations (the number of "below" classes) from the above medical threshold

above = shuffled_df9m.loc[shuffled_df9m["ADH_BELOW_THRESH9m"] == 0].sample(n=len(below),random_state=1)

# keep the samples non-selected aside (for future testing)

not_sel = shuffled_df9m[(shuffled_df9m["ADH_BELOW_THRESH9m"] == 0) & (~shuffled_df9m.index.isin(set(above.index)))]

# Concatenate "above" and "below" sampled dataframes and shuffle again

undersampled_df9m = pd.concat([below, above]).sample(frac=1, random_state=1)

#plot the dataset after the undersampling

plt.figure(figsize=(8, 8))

sns.countplot("ADH_BELOW_THRESH9m", data=undersampled_df9m)

plt.title("Balanced Classes")

plt.xlabel("Adherence below threshold")

plt.ylabel("Count")

plt.show()

print("undersampled dataframe size: ",undersampled_df9m.shape)

print("remainder dataset of class 0 (above): ", not_sel.shape)

# split data into train (for training and optimizing models in a 5-fold cross-validation scheme) and test set (for assesing model performance)

X = undersampled_df9m.drop("ADH_BELOW_THRESH9m", axis=1)

y = undersampled_df9m["ADH_BELOW_THRESH9m"]

X9m_tr, X_te, Y9m_tr, y_te = train_test_split(X, y, test_size=0.2, random_state=0)

# creating a new test set that follows the 22% - 78% distribution by completing as needed with "above" samples not selected previously

total_needed = int(np.ceil(len(y_te[y_te == 1]) / dist_full_data[1])) #round to the next integer

above_toadd = total_needed - len(y_te)

sample_toadd = not_sel.sample(above_toadd, random_state=0)

X_te_full9m = pd.concat([X_te, sample_toadd.drop("ADH_BELOW_THRESH9m", axis=1)], axis=0)

y_te_full9m = pd.concat([y_te, sample_toadd["ADH_BELOW_THRESH9m"]], axis=0)

print("Training size: ", X9m_tr.shape)

print("matrix Test size: ", X_te_full9m.shape)

print("target test size: ", y_te_full9m.shape)

#undersampled dataframe size: (4838, 11)

#remainder dataset of class 0 (above): (6091, 11)

#Training size: (3870, 10)

#matrix Test size: (2214, 10)

#target test size: (2214,)

# proportion of examples 0 and 1 in the train set

Y9m_tr.value_counts(normalize=True).round(3)*100

Y9m_tr.value_counts()

# proportion of examples 0 and 1 in the test set

y_te_full9m.value_counts(normalize=True).round(3)*100

y_te_full9m.value_counts()

y_te_full9m.shape

# create a dictionary to store optimized algorithms

best_model9m = {}

# score to optimize for

scoring = {

"f1" : "f1" #

}

#random forest

rf = RandomForestClassifier(random_state=1)

# Create cross-validation object

grid_cv = GridSearchCV(rf, {

"n_estimators": [100,300,500,700,900],

"bootstrap": [True]

}, scoring=scoring, cv=5, refit=False)

# Fit estimator

grid_cv.fit(X9m_tr, Y9m_tr)

# Collect results in a DataFrame

df9m_rf = pd.DataFrame.from_dict(dict(

{"n_estimators": grid_cv.cv_results_["param_n_estimators"],

"bootstrap" : grid_cv.cv_results_["param_bootstrap"],

"mean_f1": grid_cv.cv_results_["mean_test_f1"],

"std_f1": grid_cv.cv_results_["std_test_f1"]

}))

df9m_rf.sort_values(by=["mean_f1"], ascending=False).head(5).round(3)

best_model9m["RF_BS_T_300"] = 0.780

#Extra trees

xtc = ExtraTreesClassifier(random_state=1)

grid_cv = GridSearchCV(xtc, {

"n_estimators": [500, 600, 800, 900]

}, scoring=scoring, cv=5, refit=False)

# Fit estimator739

grid_cv.fit(X9m_tr, Y9m_tr)

# Collect results in a DataFrame

df9m_xtc = pd.DataFrame.from_dict(dict(

{"n_estimators": grid_cv.cv_results_["param_n_estimators"],

"mean_f1": grid_cv.cv_results_["mean_test_f1"],

"std_f1": grid_cv.cv_results_["std_test_f1"],

}))

df9m_xtc.sort_values(by=["mean_f1"], ascending=False).head(10).round(3)

best_model9m["ExtraTrees_600"] = 0.771

#Logistic regression

logreg = LogisticRegression(solver="saga", random_state=1, max_iter=5000)

# Create pipeline

pipe = Pipeline([

("scaler", None), # Optional step

("logreg", logreg)

])

# Create cross-validation grid search object

grid_cv = GridSearchCV(pipe, [{

"scaler" : [None, MinMaxScaler(), StandardScaler(), RobustScaler()],

"logreg__penalty" : ["l1", "l2"],

"logreg__C": np.logspace(-2,2,5)

}], scoring=scoring, cv=5, refit=False)

# Fit estimator

grid_cv.fit(X9m_tr, Y9m_tr)

# Collect results in a DataFrame

df9m_logreg = pd.DataFrame.from_dict(dict(

{

"param_scaler" : grid_cv.cv_results_["param_scaler"],

"penalty" : grid_cv.cv_results_["param_logreg__penalty"],

"C" : grid_cv.cv_results_["param_logreg__C"],

"mean_f1": grid_cv.cv_results_["mean_test_f1"],

"std_f1": grid_cv.cv_results_["std_test_f1"],

}))

df9m_logreg.sort_values(by=["mean_f1"], ascending=False).head(5).round(3)

best_model9m["LogReg_RS_l2_C100"] = 0.768

#ordinal logistic regression

olg = mord.LogisticIT()

pipe = Pipeline([

("scaler", None), # Optional step

("olg", olg)

])

# Create cross-validation object

grid_cv = GridSearchCV(pipe, {

"scaler" : [None, StandardScaler(), MinMaxScaler(), RobustScaler()],

"olg__alpha": [.1, 0.3, 1, 3, 10, 30, 100, 300]

}, scoring=scoring, cv=5, refit=False)

# Fit estimator

grid_cv.fit(X9m_tr, Y9m_tr.astype("int"))

# Collect results in a DataFrame

df9m_olr = pd.DataFrame.from_dict(dict(

{

"scaler" : grid_cv.cv_results_["param_scaler"],

"alpha": grid_cv.cv_results_["param_olg__alpha"],

"mean_f1": grid_cv.cv_results_["mean_test_f1"],

"std_f1": grid_cv.cv_results_["std_test_f1"],

}))

df9m_olr.sort_values(by=["mean_f1"], ascending=False).head(5).round(3)

best_model9m["OLR_None_alpha_30"] = 0.768

#optimized algorithms

best_model9m = pd.DataFrame.from_dict(best_model9m, orient="index", columns=["F1"])

best_model9m.sort_values(by=["F1"], ascending=False)

model9m = RandomForestClassifier(n_estimators=300, bootstrap=True, random_state=1)

# plot ROC curves on 5-fold CV on the train set

from sklearn.metrics import auc

from sklearn.metrics import plot_roc_curve

# Classification and ROC analysis

# Run classifier with cross-validation and plot ROC curves

cv = StratifiedKFold(n_splits=5, shuffle=True, random_state=1)

classifier9m = model9m

Xvals = X9m_tr.values

yvals = Y9m_tr.values

tprs = []

aucs = []

mean_fpr = np.linspace(0, 1, 100)

sns.set(style="whitegrid")

fig, ax = plt.subplots(figsize=(8,5))

for i, (train, test) in enumerate(cv.split(Xvals, yvals)):

classifier9m.fit(Xvals[train], yvals[train])

viz = plot_roc_curve(classifier9m, Xvals[test], yvals[test],

name="ROC fold {}".format(i),

alpha=0.3, lw=1, ax=ax)

interp_tpr = np.interp(mean_fpr, viz.fpr, viz.tpr)

interp_tpr[0] = 0.0

tprs.append(interp_tpr)

aucs.append(viz.roc_auc)

ax.plot([0, 1], [0, 1], linestyle="--", lw=2, color="r",

label="Chance", alpha=.8)

mean_tpr = np.mean(tprs, axis=0)

mean_tpr[-1] = 1.0

mean_auc = auc(mean_fpr, mean_tpr)

std_auc = np.std(aucs)

ax.plot(mean_fpr, mean_tpr, color="b",

label=r"Mean ROC (AUC = %0.2f $\pm$ %0.2f)" % (mean_auc, std_auc),

lw=2, alpha=.8)

std_tpr = np.std(tprs, axis=0)

tprs_upper = np.minimum(mean_tpr + std_tpr, 1)

tprs_lower = np.maximum(mean_tpr - std_tpr, 0)

ax.fill_between(mean_fpr, tprs_lower, tprs_upper, color="grey", alpha=.2,

label=r"$\pm$ 1 std. dev.")

ax.set(xlim=[-0.05, 1.05], ylim=[-0.05, 1.05],

title="Receiver operating characteristic - train set, 5 fold cross-validation")

ax.legend(loc="lower right")

plt.show()

# plot ROC curve on test set

from sklearn.metrics import auc

from sklearn.metrics import plot_roc_curve

classifier9m = model9m

classifier9m.fit(X9m_tr, Y9m_tr)

sns.set(style="whitegrid")

fig, ax = plt.subplots(figsize=(8,5))

viz = plot_roc_curve(classifier9m, X_te_full9m, y_te_full9m,

name="ROC", color="b",

alpha=1, lw=2, ax=ax)

ax.plot([0, 1], [0, 1], linestyle="--", lw=2, color="r",

label="Chance", alpha=.8)

ax.set(xlim=[-0.05, 1.05], ylim=[-0.05, 1.05],

title="Receiver operating characteristic - test set (22% - 78% class distribution)")

ax.legend(loc="lower right")

plt.savefig("/plots/roc_curve_test9months.png", dpi=300)

plt.show()

plt.savefig("/plots/roc_curve_test9months.eps", format='eps')

plt.show()

# Metric on the test set - balanced

model9m.fit(X9m_tr, Y9m_tr)

model9m_preds = model9m.predict(X_te_full9m)

tn, fp, fn, tp = confusion_matrix(y_te_full9m, model9m_preds).ravel()

sensitivity = tp / (tp+fn)

specificity = tn / (tn+fp)

print(f"The sensitivity (recall) of the model is : {sensitivity:.2f}")

print(f"The specificity (true negative rate) of the model is : {specificity:.2f}")

print(f"The f1-score of the model is : {metrics.f1_score(y_te_full9m, model9m_preds):.2f}")

#The sensitivity (recall) of the model is : 0.72

#The specificity (true negative rate) of the model is : 0.80

#The f1-score of the model is : 0.60

# confusion matrix on test set - normalized

#fig, ax = plt.subplots(figsize=(8,8))

sns.set(style="white")

plot_confusion_matrix(model9m, X_te_full9m, y_te_full9m, normalize="true")

plt.xticks(np.arange(2), ["Above 85%", "Below 85%"])

plt.yticks(np.arange(2), ["Above 85%", "Below 85%"])

plt.title("Confusion matrix normalized\nRandom Forest model", loc = "left")

plt.savefig("/plots/confusion_matrix_normalized9months.png", dpi=300, bbox_inches="tight")

# plot confusion matrix with absolute numbers

sns.set(style="white")

plot_confusion_matrix(model9m, X_te_full9m, y_te_full9m, values_format='d')

plt.xticks(np.arange(2), ["Above 85%", "Below 85%"])

plt.yticks(np.arange(2), ["Above 85%", "Below 85%"])

plt.title("Confusion matrix\nRandom Forest model", loc = "left")

plt.savefig("/plots/confusion_matrix9months.png", dpi=300, bbox_inches="tight")

plt.savefig("/plots/confusion_matrix9months.eps", format='eps')

plt.savefig("/plots/confusion_matrix9months.png", format='png')

#Comparison with a simple baseline model (simple heurestics) based on mean adherence in first months only

# simple model applied only the test set (for 1 to 1 comparison with the ML performances)

comp = X_te_full9m.copy()

comp["Simple_pred"] = np.where(comp["ADH_MEAN"]>85, 0, 1)

y_simple_pred = comp["Simple_pred"]

tn_simple, fp_simple, fn_simple, tp_simple = confusion_matrix(y_te_full9m, y_simple_pred).ravel()

sensitivity_simple = tp_simple / (tp_simple+fn_simple)

specificity_simple = tn_simple / (tn_simple+fp_simple)

print(f"The sensitivity (recall) of the simple heuristics model is : {sensitivity_simple:.2f}")

print(f"The specificity (true negative rate) of the simple heuristics model is : {specificity_simple:.2f}")

print(f"The f1-score of the simple heuristics model is : {metrics.f1_score(y_te_full9m, y_simple_pred):.2f}")

#The sensitivity (recall) of the simple heuristics model is : 0.39

#The specificity (true negative rate) of the simple heuristics model is : 0.97

#The f1-score of the simple heuristics model is : 0.52

cm = confusion_matrix(y_te_full9m, y_simple_pred, normalize="true")

plt.figure(figsize=(5,4))

g = sns.heatmap(

cm,

cmap="viridis",

vmin=0.2, vmax=0.8,

annot=True,

xticklabels=["Above 85%", "Below 85%"],

yticklabels=["Above 85%", "Below 85%"])

g.set_yticklabels(g.get_yticklabels(), rotation = 0)

plt.xlabel("Predicted label")

plt.ylabel("True label")

plt.title("Confusion matrix normalized\nsimple heuristic mode", loc = "left")

plt.savefig("/plots/confusion_matrix_normalized_simple_heuristics9months.png", dpi=300, bbox_inches="tight")

cm = confusion_matrix(y_te_full9m, y_simple_pred)

plt.figure(figsize=(5,4))

g = sns.heatmap(

cm,

cmap="viridis",

vmin=100, vmax=1500,

annot=True,

fmt="d",

xticklabels=["Above 85%", "Below 85%"],

yticklabels=["Above 85%", "Below 85%"])

g.set_yticklabels(g.get_yticklabels(), rotation = 0)

plt.xlabel("Predicted label")

plt.ylabel("True label")

plt.title("Confusion matrix normalized\nsimple heuristic model", loc = "left")

plt.savefig("/plots/confusion_matrix_simple_heuristics9months.png", dpi=300, bbox_inches="tight")

# comparing f1-scores

f1_ml = metrics.f1_score(y_te_full9m, model9m_preds)

f1_simple = metrics.f1_score(y_te_full9m, y_simple_pred)

pd.DataFrame(data=[f1_ml, f1_simple], columns=["F1 score"], index=["ML Model", "Simple Model"]).round(3)

# F1 score

#ML Model 0.599

#Simple Model 0.520

# plot ROC curve on test set (2 plots)

from sklearn.metrics import auc

from sklearn.metrics import plot_roc_curve

classifier9m = model9m

onlyadh9m = classifier9m.fit(

X9m_tr[["ADH_MEAN", "ADH_STD"]] #.values.reshape(-1,1)

,Y9m_tr

)

sns.set(style="whitegrid")

fig, ax = plt.subplots()

viz9m = plot_roc_curve(onlyadh9m, X_te_full9m[["ADH_MEAN", "ADH_STD"]] #.values.reshape(-1,1)

, y_te_full9m,

name="ROC Adherence only", color=m_darkblue,

alpha=1, lw=2, ax=ax)

allfeat9m = classifier9m.fit(X9m_tr, Y9m_tr)

viz29m = plot_roc_curve(allfeat9m, X_te_full9m, y_te_full9m,

name="ROC All features", color=m_darkgreen,

alpha=1, lw=2, ax=ax)

ax.plot([0, 1], [0, 1], linestyle="--", lw=2, color="r",

label="Chance", alpha=.8)

ax.set(xlim=[-0.05, 1.05], ylim=[-0.05, 1.05],

title="Receiver operating characteristic - test set")

ax.legend(loc="lower right")

plt.savefig("/plots/roc_curve_comp_onlyadh9months.png", dpi=300)

plt.show()

#Machine learning model interpretation

explainer = shap.TreeExplainer(model9m)

shap_values = explainer.shap_values(X_te_full9m)

# feature importance plot; importance is ordered by descreasing

shap.summary_plot(shap_values[1], X_te_full9m, plot_type="bar")

shap.summary_plot(shap_values[1], X_te_full9m, show=False)

plt.savefig("/plots/shap_plot9months.png", dpi=300, bbox_inches="tight")

plt.savefig("/plots/shap_plot9months.eps", format='eps')

#interpretation with LIME

# rename columns (shorter names needed)

X9m_tr_renamed = X9m_tr.copy().rename({

"AGE_START":"age", # age at start of use

"START_MONTH":"mstart", # month at start of use

"ADH_MEAN":"adh_mean", # mean adherence over the first 3 months

"ADH_STD":"adh_std", # adherence standard deviation over the first 3 months

"NR_TRANS":"nr_trans", # nr of transmissions in the first 3 months

"INJECTION_TIME":"itime", # injection time setting (s)

"INJECTION_DEPTH":"idepth", # injection depth setting (mm)

"INJECTION_SPEED" : "ispeed", # injection speed setting (0:slow, 1:medium, 2:fast)

"NEEDLE_SPEED":"nspeed", # needle speed setting (0:slow, 1:medium, 2:fast)

"NR_DOSE_CHANGES": "dosech", # number of dose changes in the first 3 months

"GENDER" : "gender" # gender (0: girl, 1:boy)

}, axis=1)

#random sampling 10 instance where the real adherence to be predicted should be "below", respectively "above"

below_explain = y_te_full9m[y_te_full9m == 1].sample(10, random_state=1).index.tolist()

above_explain = y_te_full9m[y_te_full9m == 0].sample(10, random_state=1).index.tolist()

# set up and fit lime model explainer

predict_model9m = lambda x: model9m.predict_proba(x).astype(float)

explainer_model9m = lime.lime_tabular.LimeTabularExplainer(

X9m_tr.values,

feature_names = X9m_tr_renamed.columns,

categorical_features=["boy"],

class_names=["Above","Below"],

kernel_width=5

)

# explaining class "below 85%"

for instance in below_explain:

print("real class: below 85% adherence")

choosen_instance = X_te_full9m.loc[[instance]].values[0]

display(X_te_full9m.loc[[instance]])

print("interpretation")

exp_model9m = explainer_model9m.explain_instance(choosen_instance, predict_model9m, num_features=11)

exp_model9m.show_in_notebook(show_all=True)

exp_model9m.save_to_file(f"instance_below_{instance}.html")

# explaining class 0

for instance in above_explain:

choosen_instance = X_te_full9m.loc[[instance]].values[0]

display(X_te_full9m.loc[[instance]])

print("interpretation")

exp_model9m = explainer_model9m.explain_instance(choosen_instance, predict_model9m, num_features=11)

exp_model9m.show_in_notebook(show_all=True)

exp_model9m.save_to_file(f"instance_above_{instance}.html")

# make a copy of the dataframe for hypothesis testing purposes

df9m_hypothesis = undersampled_df9m.copy()

# bin features in categories as defined above

df9m_hypothesis["ADH_BELOW_THRESH9m"] = np.where(df9m_hypothesis["ADH_BELOW_THRESH9m"]==1, "below thresh.", "above thresh.")

df9m_hypothesis["AGE_START"] = np.where(df9m_hypothesis["AGE_START"]>10, ">10", "<=10")

df9m_hypothesis["GENDER"] = np.where(df9m_hypothesis["GENDER"]==1, "Boy", "Girl")

df9m_hypothesis["NR_TRANS"] = np.where(df9m_hypothesis["NR_TRANS"]==0, "0", ">0")

df9m_hypothesis["ADH_MEAN"] = np.where(df9m_hypothesis["ADH_MEAN"]< 90, "<90", ">=90")

df9m_hypothesis["ADH_STD"] = np.where(df9m_hypothesis["ADH_STD"]>9, ">9", "<=9")

df9m_hypothesis["NEEDLE_SPEED"] = np.where(df9m_hypothesis["NEEDLE_SPEED"]<=1, "low/medium", "fast")

df9m_hypothesis["INJECTION_TIME"] = np.where(df9m_hypothesis["INJECTION_TIME"]<=5, "<=5", ">5")

df9m_hypothesis["NR_DOSE_CHANGES"] = np.where(df9m_hypothesis["NR_DOSE_CHANGES"] == 0, "0", ">0")

df9m_hypothesis["INJECTION_DEPTH"] = np.where(df9m_hypothesis["INJECTION_DEPTH"]>6, "<=6", ">6")

# perform chi2 statistical tests for each of the features binned previously

stats_p_values = {}

for column in df9m_hypothesis.drop(["ADH_BELOW_THRESH9m", "INJECTION_SPEED"], axis=1).columns:

ct = pd.crosstab(df9m_hypothesis[column], df9m_hypothesis["ADH_BELOW_THRESH9m"])

chi2, p, dof, ex = chi2_contingency(observed=ct)

stats_p_values[column] = [chi2, p]

# gather test results (feature and associated p-value in a dataframe)

df9m_tests = pd.DataFrame.from_dict(stats_p_values, orient="index", columns=["Chi2", "p_value"]).reset_index().sort_values(by="p_value", ascending=True)

df9m_tests = df9m_tests.rename({"index":"Feature"}, axis=1)

# display all results ordered by p-value in ascending order

display(df9m_tests.drop("Chi2", axis=1).round(3))

# only display results for which the p-value is below 0.05 significance threshold

display(df9m_tests[df9m_tests["p_value"]<0.05].drop("Chi2", axis=1))

# recalculate the bonferroni corrected p-value

# (accounting for the fact that sample size may differ and that multiple tests are performed)

reject, p_adjusted, _, _ = multipletests(df9m_tests["p_value"], method="bonferroni")

# gather bonferroni corrected p-values in a dataframe

df9m_tests_bc = (

pd.DataFrame(

list(zip(df9m_tests["Feature"].values, p_adjusted, reject)),

columns=["Feature", "bf-corrected p_value", "Reject"]

)

.sort_values(by="bf-corrected p_value", ascending=True).round(3)

)

df9m_tests_bc = df9m_tests_bc.rename({"index":"Feature"}, axis=1)

# display only statistically significant features under a bonferroni corrected p-value

display(df9m_tests_bc[df9m_tests_bc["Reject"] == True])

#####################ROC curve

# plot ROC curve on test set (3 model plots)

classifier3m = model3m

classifier6m = model6m

classifier9m = model9m

sns.set(style="whitegrid")

fig, ax = plt.subplots()

allfeat3m = classifier3m.fit(X3m_tr, Y3m_tr)

allfeat6m = classifier6m.fit(X6m_tr, Y6m_tr)

allfeat9m = classifier9m.fit(X9m_tr, Y9m_tr)

viz23m = plot_roc_curve(allfeat3m, X_te_full3m, y_te_full3m,

name="ROC following 3 months", color="r",

alpha=1, lw=2, ax=ax)

viz26m = plot_roc_curve(allfeat6m, X_te_full6m, y_te_full6m,

name="ROC following 6 months", color=m_darkblue,

alpha=1, lw=2, ax=ax)

viz29m = plot_roc_curve(allfeat9m, X_te_full9m, y_te_full9m,

name="ROC following 9 months", color=m_darkgreen,

alpha=1, lw=2, ax=ax)

ax.plot([0, 1], [0, 1], linestyle="--", lw=2, color="r",

label="Chance", alpha=.8)

ax.set(xlim=[-0.05, 1.05], ylim=[-0.05, 1.05],

title="Receiver operating characteristic - test set")

ax.legend(loc="lower right")

figure = plt.gcf()

figure.set_size_inches(6, 6)

plt.savefig("/plots/roc_curve_comp_onlyadh369months.png", dpi=300)

plt.show()
